# Effects of vibration training on motor and non-motor symptoms for patients with multiple sclerosis: A systematic review and meta-analysis

**DOI:** 10.3389/fnagi.2022.960328

**Published:** 2022-08-05

**Authors:** Yang Zhang, Peng Xu, Yu Deng, Wenxiu Duan, Juncai Cui, Chaomin Ni, Ming Wu

**Affiliations:** Department of Rehabilitation Medicine, The First Affiliated Hospital of USTC, Division of Life Sciences and Medicine, University of Science and Technology of China, Hefei, China

**Keywords:** vibration therapy, multiple sclerosis, motor and non-motor symptoms, meta-analysis, physical therapy

## Abstract

**Background:**

Vibration therapy is one of the rehabilitation programs that may be effective in treating both motor and non-motor symptoms in Multiple Sclerosis patients. We conducted a comprehensive systematic review and meta-analysis to assess the effects of vibration therapy on motor and non-motor symptoms (functional mobility, balance, walking endurance, gait speed, fatigue, and quality of life) of this population.

**Methods:**

A systematic search of PubMed, Embase, the Cochrane Library, Web of Science, Physiotherapy Evidence Database, Scopus, Google Search Engine, and the China National Knowledge Infrastructure (CNKI). Two reviewers independently assessed the study quality.

**Results:**

Fourteen studies with 393 participants were finally included in the meta-analysis. The pooled results showed that vibration therapy had a significant advantage over the control intervention in improving balance function [mean difference (MD) = 2.04, 95% confidence interval (CI): 0.24–3.84, *P* = 0.03], and walking endurance (SMD = 0.34, 95% CI: 0.07–0.61, *P* = 0.01). Meanwhile, the degree of disability subgroup analysis revealed that the Expanded Disability Status Scale (EDSS) score (3.5–6) significantly improved functional mobility (MD: −1.18, 95% CI: −2.09 to 0.28, *P* = 0.01) and balance function (MD: 3.04, 95% CI: 0.49–5.59, *P* = 0.02) compared with the control group, and the EDSS (0–3.5) were more beneficial in walking endurance. The duration subgroup analysis indicated a significant difference in the effect of the duration (<4 weeks) on enhancing walking endurance (SMD: 0.46, 95% CI: 0.04–0.87, *P* = 0.03). However, no significant improvement was found in functional mobility, gait speed, fatigue, and quality of life.

**Conclusion:**

Vibration therapy may improve balance function and walking endurance, and the degree of disability and duration of intervention may affect outcomes. The evidence for the effects of vibration therapy on functional mobility, gait speed, fatigue, and quality of life remains unclear. More trials with rigorous study designs and a larger sample size are necessary to provide this evidence.

**Systematic Review Registration:**

PROSPERO, https://www.crd.york.ac.uk/prospero/#recordDetails, identifier: CRD42022326852.

## Introduction

Multiple sclerosis (MS) is an inflammatory, demyelinating disease of the central nervous system (CNS), with neurodegeneration being most prominent in progressive phenotypes (Benedict et al., 2020). In 2016, about 2.2 million people were affected globally, with rates varying widely in different regions and among different populations (Wallin et al., 2019). The disease usually begins between the ages of 20 and 50 and is twice as common in women as in men (Milo and Kahana, 2010). The symptoms and dysfunction of MS include muscle weakness, sensory, balance, and mobility problems, spasticity, tremor, rapidly growing fatigue, or cognitive difficulties, which significantly influence the quality of life among MS patients (Compston and Coles, 2008; Benedict et al., 2020; Zielinska-Nowak et al., 2020). Despite disease-modifying therapies (DMTs) reducing the rate of disease progression (Hauser and Cree, 2020), the development of effective rehabilitation programs remain essential in managing the disease (Zuber et al., 2020).

Vibration therapy is one of the rehabilitation programs that have potential benefits on muscle performance, mobility, postural control, and proprioception in healthy and neurological populations (Alashram et al., 2019a; Moggio et al., 2021). It takes advantage of sinusoidal mechanical oscillation to stimulate muscles, characterized by amplitude, frequency, and phase angle, that might be used in the rehabilitation field as whole-body vibration (WBV) and focal muscle vibration (FMV) (Alashram et al., 2019b; Moggio et al., 2021). Numerous mechanisms have been proposed to explain the vibration phenomenon (Cochrane, 2011). The transmission of vibrations and oscillations to the biological system can produce physiological changes on several levels by stimulating skin receptors (Sonza et al., 2013), muscle spindles (Barrera-Curiel et al., 2019), and vestibular system (Ardic et al., 2021). The most common hypothesis is that vibration can affect the muscle spindle and lead to increased α-motor neurons and enhance muscle contraction (Cardinale and Bosco, 2003; Abercromby et al., 2007; Rittweger, 2010).

In the past decades, several studies used different parameters and treatment protocols of vibration therapy have reported improvements in muscle strength, functional mobility, balance, spasticity, fatigue, and participation in activities of daily living in subjects with MS (Schuhfried et al., 2005; Broekmans et al., 2010; Claerbout et al., 2012; Paoloni et al., 2013; Uszynski et al., 2014; Ebrahimi et al., 2015; Spina et al., 2016). However, other studies did not show improvements (Wolfsegger et al., 2014; Freitas et al., 2018; Ayvat et al., 2021). Six review articles (Santos-Filho et al., 2012; Sitja Rabert et al., 2012; Kantele et al., 2015; Kang et al., 2016; Castillo-Bueno et al., 2018; Alam et al., 2020) examine the impact of WBV on patients with MS have been published. A 2012 Cochrane Review found no evidence of a short-term or long-term effect of WBV on any functional outcomes (body balance, gait, muscle performance) or QoL, compared with other active physical therapy or passive intervention (Sitja Rabert et al., 2012). The authors recommended further investigation given the limitations of the review, with only four low-quality trials included. More recently, a systematic review and meta-analysis included 8 RCTs that showed an overall effect of WBV on strength and some measures of balance and mobility, but its impact remains inconclusive (Alam et al., 2020). Furthermore, all review articles included studies that were published in 2015 or earlier, and only focused on the WBV. Meanwhile, no reviews have been established to focus on the impacts of vibration therapy on non-motor impairments such as quality of life, disability level, or fatigue after MS, and the influence of stimulation parameters, including frequency and duration, was not evaluated.

In light of the limitations of these prior reviews, the clinical conclusion to date was that there was insufficient high-level evidence to support the routine use of vibration therapy for improving both motor and non-motor impairments, and the potential for vibration therapy to improve access to, and quality of, rehabilitation services while reducing costs, an update of the previous review was warranted. Besides, more studies have been carried out in recent years due to the further development and appliance of vibration therapy (Paoloni et al., 2013; Ebrahimi et al., 2015; Spina et al., 2016; Uszynski et al., 2016; Ayvat et al., 2021). Hence, the purposes of this review were to examine the effects of vibration therapy on motor and on-motor impairments–focused outcomes in individuals with MS and to investigate which vibration exposure parameters (i.e., frequency, EDSS, duration) induced improvement in motor and non-motor symptoms.

## Materials and Methods

This systematic review and meta-analyses were conducted according to the Preferred Reporting Items for Systematic Reviews and Meta-Analyses (PRISMA) statement and by use of applying research protocol (Moher et al., 2009).

### Search strategy

Data were collected from PubMed, Embase, the Cochrane Library, Web of Science, Physiotherapy Evidence Database, Scopus, Google Search Engine, and the China National Knowledge Infrastructure (CNKI). These databases were searched systematically from inception to December 2021. In addition, reference lists of identified studies were also screened to identify additional relevant articles. The keywords were entered using a standard search and included “whole-body vibration,” “WBV,” “vibration,” “focal vibration,” “focal vibration therapy,” “focal muscle vibration,” “localized vibration,” “FVT,” “FMV,” and “multiple sclerosis.” There was no restriction on language. Two reviewers initially evaluated the obtained studies by reading the title and abstract to exclude studies that did not meet the criteria. They then read the full text to determine eligibility. The detailed search strategy is described in Appendix 1 in Supplementary material.

### Inclusion and exclusion criteria

Studies were selected based on the following inclusion criteria: (1) Study design: Randomized controlled trials (RCTs); (2) Patients: patients diagnosed with MS, without age or level of disability restrictions; (3) Intervention: vibration therapy, WBV or FMV; (4) Control: placebo, sham treatment, exercise alone or conventional rehabilitation; (5) Outcomes: at least one outcome related to either motor and non-motor performance was provided. The motor performance index includes Timed Up and Go (TUG), Berg Balance Scale (BBS), 6-minute walk test (6MWT), 2-minute walk test (2MWT); 3MWT: 3-minute walk test (3MWT), 10-m walk test (10MWT), and Timed 25-foot Walk (T25FW). The TUG is an excellent tool used to assess mobility capabilities in MS, with good reliability (ICC = 0.98) (Kalron et al., 2017; Valet et al., 2019). The minimal detectable change (MDC95) was 1.3 s (Valet et al., 2019). The BBS has been confirmed, test-retest and inter-rater reliability were excellent in MS (ICC > 0.95) (Cattaneo et al., 2007), and there is strong first-level evidence to support its use to assess changes in static and dynamic sitting balance (Moore et al., 2018; Mehta et al., 2019).

The 6MWT and 2MWT have high reliability (ICC: 0.95–0.99) in persons with MS and are responsive to changes in deteriorating status in persons with MS (Fry and Pfalzer, 2006; Bennett et al., 2017). The 10MWT and 25FWT provide a performance-based measure of walking dysfunction based on walking speed over a short distance, with greater disability of MS with a very high correlation (0.96–0.97) (Kieseier and Pozzilli, 2012). The non-motor performance index includes the Fatigue Severity Scale (FSS), Modified Fatigue Impact Scale (MFIS), Multiple Sclerosis Quality of Life-54 (MSQOL-54), and Multiple Sclerosis Impact Scale (MSIS-29). The FSS and MFIS can be regarded as feasible measures of self-reported fatigue in MS. The data quality of the FSS was excellent, with 99.6% of computable scale scores and floor and ceiling effects were minimal (Rosti-Otajarvi et al., 2017). The reported internal consistency of all the MFIS scores was “excellent,” with the following Cronbach α values: total, 0.81; cognitive, 0.95; physical, 0.91; and psychosocial, 0.81 and a change in score of 10 or more to be clinically relevant (Kos et al., 2007). The MSQOL-54 is a structured, self-report questionnaire that the patient can generally complete with little or no assistance with the alpha coefficient calculated for the whole instrument was 0.84 (Heiskanen et al., 2007).

The MSIS-29 is a new patient-based rating scale for multiple sclerosis (MS) that was predominantly developed from a community-based sample derived from the MS Society. Floor and ceiling effects were small and were considerably less than the recommended maximum of 15% (Riazi et al., 2002). The lowest score was 3.8 and the Cronbach's α exceeded the standard criteria of 0.80 (Riazi et al., 2002).

Exclusion criteria were as follows: (1) retrospective studies, animal studies, single-case reports, protocols, reviews, meta-analyses, poster presentations, or conference abstracts; (2) study objective or intervention measures failed to meet the inclusion criteria; (3) duplicate or multiple publications of the same study; and (4) studies without usable data.

### Data extraction

The abstracts of retrieved studies were independently reviewed by two authors (YD and PX), and full articles were examined when necessary. The data were extracted independently by these two authors, and any disagreements were resolved by discussion with at least one more author until a consensus was reached. If more than one article was published from the same cohort, the study with the most comprehensive data was selected for inclusion. The following information was extracted from all qualifying articles: general information (name of the first author, publication year, the region where the population resided, study type, sample size, mean ages, sex), EDSS, interventions characteristics (frequency, amplitude, duration, device) and outcomes (as defined above).

### Assessment of risk of bias of included studies

The risk of bias in included trials was evaluated by two reviewers (MW and WXD) using the Cochrane Risk of Bias Tool, and another author (JCC) resolved any disagreement. The tool consists of seven elements: (1) random sequence generation; (2) allocation concealment; (3) blinding of participant and personnel; (4) blinding of outcome assessment; (5) incomplete outcome data; (6) selective reporting; (7) other bias. Every section had a high risk of bias, low risk of bias, and unclear risk of bias depending on the actual content of the included study (Higgins et al., 2022). Each of these factors was classified as high risk, low risk, or unclear risk.

### Statistical analysis

The extracted data were statistically analyzed using Review Manager software 5.3 (Cochrane Collaboration, Oxford, United Kingdom). For all the outcome measures we used, that were continuous variables, the mean difference (MD) or standardized mean difference (SMD) with a 95% confidence interval (CI) was determined. The SMD and standard error (SE) for each outcome before and after treatment were determined by Morris' formula (Morris, 2007). If the data were reported as mean and 95% CI, SD was calculated by Rev Man software. If the data were reported as the median interquartile range (IQR), we calculated the mean and standard deviation utilizing the Wan and Luo formulae (Wan et al., 2014; Luo et al., 2018). The SMD statistic was selected to evaluate the results of different scales. Cochran's Q test and Higgins I^2^ statistic were used to measure the heterogeneity of the included studies and to choose the effect model. If *I*^2^ <50% and *P* > 0.05, the included studies were considered homogeneous, and a fixed-effects model was selected. Otherwise, if *I*^2^ > 50% and *P* < 0.05, indicating that statistical heterogeneity existed among studies, a random-effects model was selected. In this meta-analysis, *P* < 0.05 was defined as statistically significant for all tests.

If clinical heterogeneity was present in the combined results, a subgroup analysis was performed to identify the source of heterogeneity. Meanwhile, we conducted subgroup analyses to explore the effect of different categories of vibration therapy. For the motor symptoms, subgroup analyses were performed for the EDSS scores, frequency, and duration.

We also performed a sensitivity analysis by sequentially eliminating each study to test the stability of the results. Before calculating the effect size, we deleted each of the included studies and excluded those that resulted in high heterogeneity or altered the pooled effect of the results (Jin et al., 2019). For the functional mobility and balance function, we used funnel plots and Egger's test to evaluate the publication bias of the included studies.

## Results

### Study identification and selection

**Figure 1:**
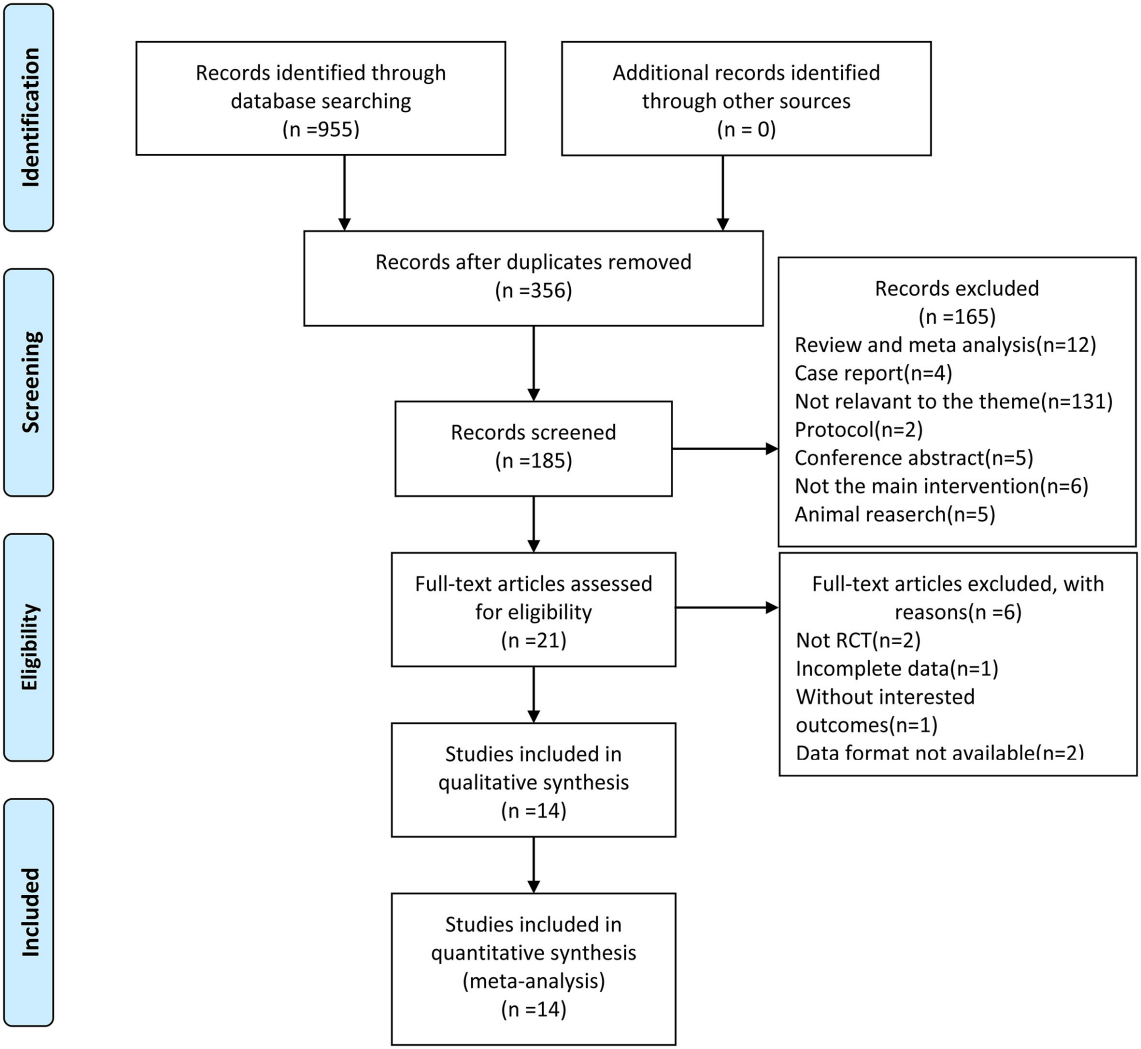
Flow of the trial selection process.

Utilizing the search strategy, a total of 955 relevant records were initially identified from the eight electronic databases. After excluding duplicates and irrelevant studies by screening titles and abstracts, 541 records were excluded. After reading the full text to identify available data, 21 were excluded. Finally, we included 14 studies (Schuhfried et al., 2005; Schyns et al., 2009; Broekmans et al., 2010; Alguacil Diego et al., 2012; Claerbout et al., 2012; Hilgers et al., 2013; Paoloni et al., 2013; Uszynski et al., 2014, 2016; Wolfsegger et al., 2014; Ebrahimi et al., 2015; Spina et al., 2016; Freitas et al., 2018; Ayvat et al., 2021) that met the criteria for data extraction and meta-analysis. The PRISMA flow diagram of identification and selection is shown in Figure 1.

### Study characteristics

The characteristics of the included studies are summarized in Table 1. A total of 14 RCTs involving 393 participants (146 males and 247 females, mean age ranged from 33.86 to 54 years) were included in the review.

**Table 1:** The characteristics of the included studies.

**References, country**	**Mean age, year**	**Duration of disease, year**	**EDSS**	**NO. T/C**	**Sex, M/F**	**Intervention group**	**Control group**	**Vibration amplitude**	**Vibration frequency**	**Duration**	**Times**	**Outcome assessments**	**Devices**
Alguacil Diego et al. (2012), Spain	T:43 (17) C:44 (20)	None	T:3.99 (0.80) C:4.58 (0.36)	17/15	16/16	WBV	None	3 mm	6 hz	5 days	5 consecutive days	BBS, TUG, 10MWT	Zeptoring^®^ vibrating platform
Ayvat et al. (2021), Turkey	T1:37.7 (9.7) T2:38.4 (11.07) C:33.86 (6.74)	T1:11.3 (6.43) T2:7 (4.74) C:10.58 (7.03)	T1:3 (1.08) T2:2.75 (1) C:3 (0.81)	11/11/11	10/23	T1:FMV (50Hz) + exercise T2:FMV (100Hz)+ exercise	Exercise	None	50 hz, 100 hz	8 weeks	3 times/week	FSS	Vibrasens©
Broekmans et al. (2010), Belgium	T:46.1 (6.96) C:49.7 (12.35)	None	T:4.5 (1.32) C:4.1 (1.12)	11/14	18/7	WBV	None	2.5 mm	25–45 hz	20 weeks	5 training sessions per 2 week cycle	BBS, TUG, 2MWT, T25FW	Alpha Vibe^®^ Nijverdal
Claerbout et al. (2012), Belgium	Tf:67.2 (14.3) Tl:43.8 (12.6) C:47.6 (8.3)	T1:12.1 (9.2) T2:12.5 (9.1) C:10.3 (8.4)	T1:5.3 (1.3) T2:5.1 (1.2) C:5.2 (1.1)	20/28/17	34/21	T1: WBV (standing on a standard mat of 2 cm thickness) + conventional therapy T2: WBV (standing on a standard mat of 10 cm thickness) + conventional therapy	Conventional therapy	1.6 mm	30–40 hz	3 weeks	10 sessions over a period of 3 weeks	BBS, TUG, 3MWT	Fysiomed NV-SA
Ebrahimi et al. (2015), Iran	T:37.06 (8.42) C:40.75 (10.56)	T:6.5 (4.17) C:10.5 (6.4)	T:3.12 (1.19) C:3.10 (0.76)	16/14	7/23	WBV	None	2 mm	2–20 hz	10 weeks	3 times/week	MFIS, BBS, 10MWT, TUG, 6MWT, MSQOL-54	None
Freitas et al. (2018), United States	46.6 (9.6)	None	None	12/9	0/21	WBV	Placebo	3 mm	30 hz	1 week	1 time/week	BBS, TUG	PowerPlate; Next Generation, Northbrook, Illinois
Hilgers et al. (2013), Germany	T:43.5 (10) C:43.9 (7.5)	None	T:3.5 (1.2) C:3.3 (1.3)	30/30	15/45	WBV	Placebo	1-2mm	30 hz	3 weeks	3 times/week	TUG, 6MWT, 10MWT	Power Plate pro
Paoloni et al. (2013), Italy	T:47.4 (5.6) C:50.6 (8.9)	None	T:4.74 (1.48) C:5.50 (1.48)	14/14	9/19	FMV and BTX	BTX	None	120 hz	4 weeks	3 times/week	FSS	Horus
Schuhfried et al. (2005), Austria	T:49.3 (13.3) C:46 (12.7)	None	T:3.9 (0.8) C:3.7 (0.8)	6/6	3/ 9	WBV	Placebo	3mm	2.0-4.4 hz	2 weeks	None	TUG	Zeptor-Med system
Schyns et al. (2009), Britain	T:45.8 (8.4) C:49.5 (6.14)	T:6.7 (5.54) C:11.8 (3.62)	None	8/8	4/12	WBV + exercise	Exercise	2mm	40hz	4 weeks	3 times/week	TUG,10 MWT, MSIS-29	VibroGym International BV
Spina et al. (2016), Italy	T:47 (12.7) C:48 (12.34)	T:7.55 (5.76) C:6.4 (8.88)	T:3.88 (1.31) C:3.7 (1.13)	10/10	8/12	FMV	Placebo	None	None	3 weeks	5 times/week	BBS, T25FW, FSS	Equistasi^®^ devices
Uszynski et al. (2014), Ireland	T:44.4 (10.4) C:52.7 (10.5)	None	None	8/9	3/14	WBV + exercise	Exercise	None	40 hz	8 weeks	3 times/week	MFIS, BBS, TUG, 6MWT, MSIS-29	Crazy Fit 1000 W 70 Speed Vibroplate
Uszynski et al. (2016), Ireland	T:45.5 (10.22) C:54 (12.22)	None	None	14/13	4/ 23	WBV + exercise	Exercise	None	40 hz	12 weeks	3 times/week	MFIS-29, 6MWT	Crazy Fit 1000W 70 Speed Vibroplate
Wolfsegger et al. (2014), Austria	T:43 (13.4) C:39.3 (10.6)	None	T:2.5 (1.0) C:2.4 (0.8)	9/8	15/2	WBV	Placebo	None	2.5–5.0 hz	3 weeks	None	TUG	Zeptor-Med system

*WBV, whole-body vibration; FMV, focal muscle vibration; TUG, Timed Up and Go; BBS, Berg Balance Scale; 6MWT, 6-minute walk test; 2MWT, 2-minute walk test; 3MWT, 3-minute walk test; 10MWT, 10-m walk test; T25FW, Timed 25-foot Walk; FSS, Fatigue Severity Scale; MFIS, Modified Fatigue Impact Scale; MSQOL-54, Multiple Sclerosis Quality of Life-54; MSIS-29, Multiple Sclerosis Impact Scale*.

The studies were published between 2005 and 2021, four (28.57%) studies were published after 2015. The countries of the publications were the Austria (*n* = 2,14.29%), Belgium (*n* = 2, 14.29%), Iran (*n* = 1, 7.14%), Italy (*n* = 2, 14.29%), Ireland (*n* = 2,14.29%), Germany (*n* = 1, 7.14%), Spain (*n* = 1, 7.14%), Britain (*n* = 1, 7.14%), United States (*n* = 1, 7.14%), and Turkey (*n* = 1, 7.14%).

The mean score of the EDSS in included studies ranged from 2.4 to 5.50. The intervention investigated in the trials included WBV (*n* = 11, 78.57%) and FMV (*n* = 3, 21.43%). The duration of the intervention in the included studies ranged from 5 days to 20 weeks. The vibration frequency of VB in the included studies ranged from 2 to 100 Hz. Twelve (85.71%) studies mentioned treatment frequency which three times weekly (*n* = 7, 50%), five times weekly (*n* = 1, 7.14%), one time weekly (*n* = 1, 7.14%), 5 consecutive days (*n* = 1, 7.14%), 5 training sessions per 2 week cycle (*n* = 1,7.14%), and 10 sessions of over a period of 3 weeks (*n* = 1,7.14%). Four (28.57%) of the control groups were treated with routine excise alone, 5 (35.71%) with placebo, 3 (21.42%) with no intervention, 1 (7.14%) with conventional therapy (multi-disciplinary), and 1 (7.14%) with botulinum toxin.

Ten (71.43%) trials selected the TUG to functional mobility. Seven (50%) trials used the BBS to assess balance. Six trials used the 6MWT (*n* = 4, 28.57%), the 2MWT (*n* = 1, 7.14%), or the 3MWT (*n* = 1) (7.14%) to assess walking endurance. Six trials used the 10MWT (*n* = 4, 28.57%) or the T25FW (*n* = 2, 14.29%) to assess gait speed. Five trials used the FSS (*n* = 2, 14.29%) or the MFIS (*n* = 3, 21.43%) to assess fatigue. Three trials used the MSQOL-54 (*n* = 1, 7.14%) or the MSIS-29 (*n* = 2, 14.29%) to assess the health-related quality of life (HRQoL).

### Risk of bias assessment

**Figure 2:**
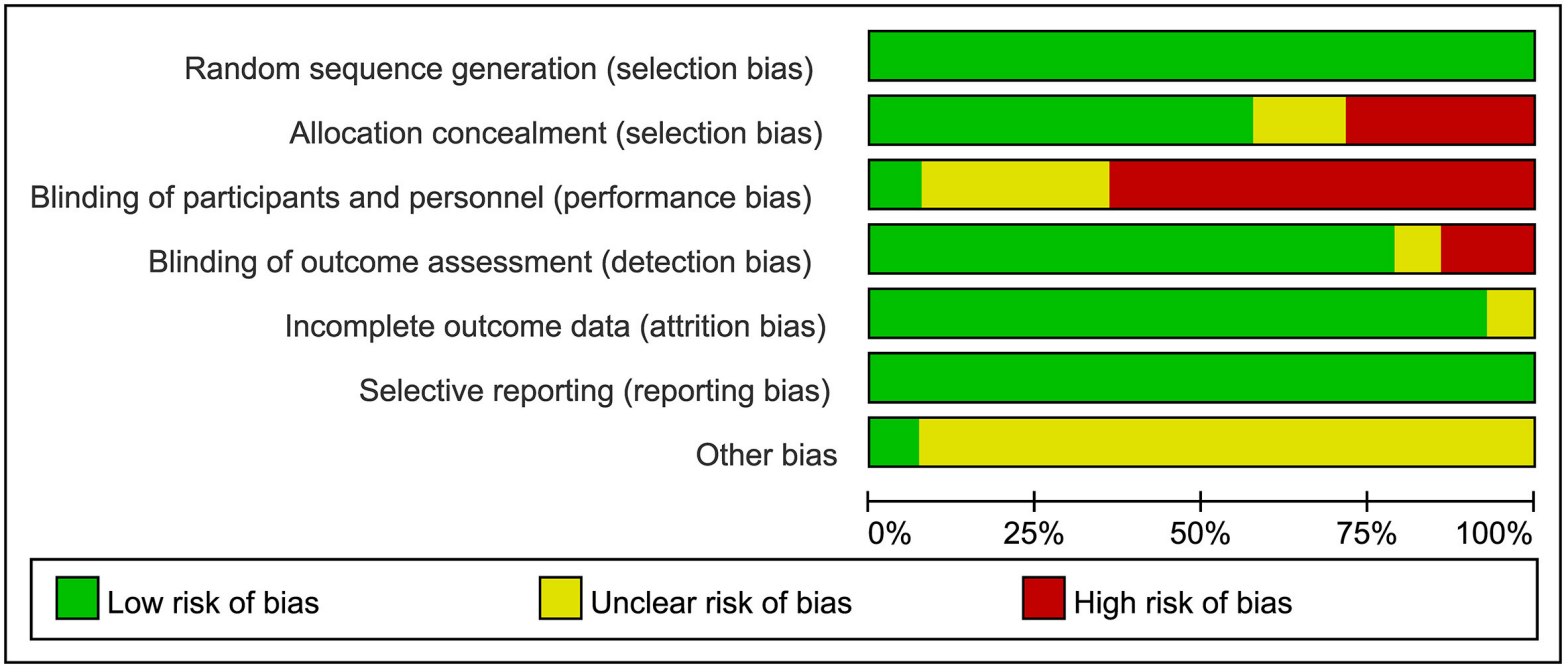
Risk of bias graph review authors' judgements about each risk of bias item presented as percentages across all included studies.

**Figure 3:**
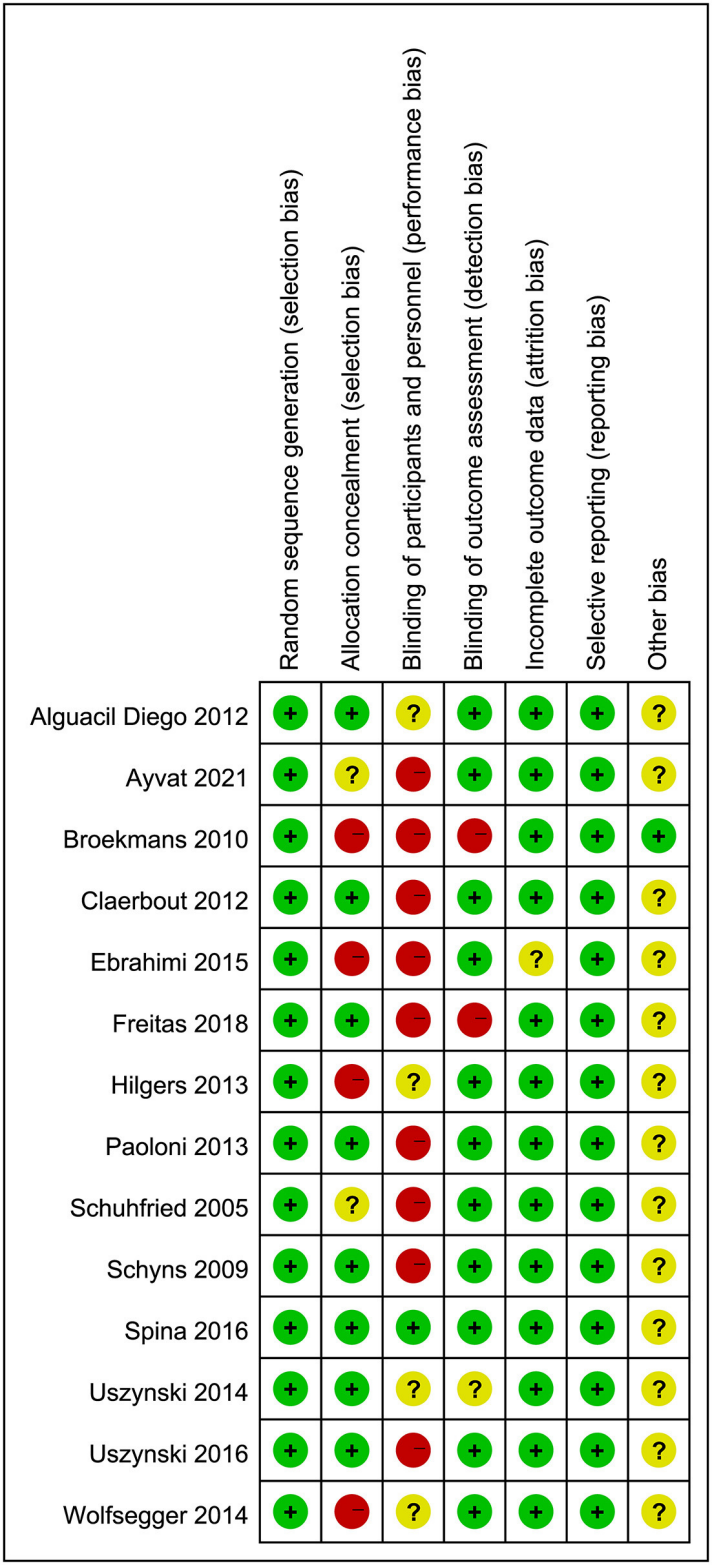
Risk of bias summary review authors' judgements about each risk of bias item for each included study.

The risk of bias assessment of the included studies is presented in Figures 2, 3. All of the included articles reported randomized group allocation, twelve studies (85.71%) reported the methods of random sequence generation, three studies (21.43%) used computer-generated random numbers, four studies (28.57%) used a random number table, three (21.43%) used envelope to allocate, one study (7.14%) used block randomization, and one study (7.14%) used gender randomization. Eight studies (57.14%) reported the use of allocation concealment. All of the included studies demonstrated a high risk of performance bias, as participants and personnel were not blind to the intervention. Only one study (7.14%) succeeded in blinding the participants and personnel. Elven studies (78.57%) were assessed as having a low risk of bias considering the blinding of assessors. Thirteen studies (92.86%) reported a low risk of attrition bias. Regarding reporting bias, we judged that all the studies reported the expected results. The risk of other bias in the included studies was judged as “unclear” due to insufficient information to judge whether there is a significant risk of bias, even if the sample size and follow-up time limitations are described.

#### Effects of the intervention

##### Motor impairments outcomes assessment

###### Functional mobility

**Figure 4:**
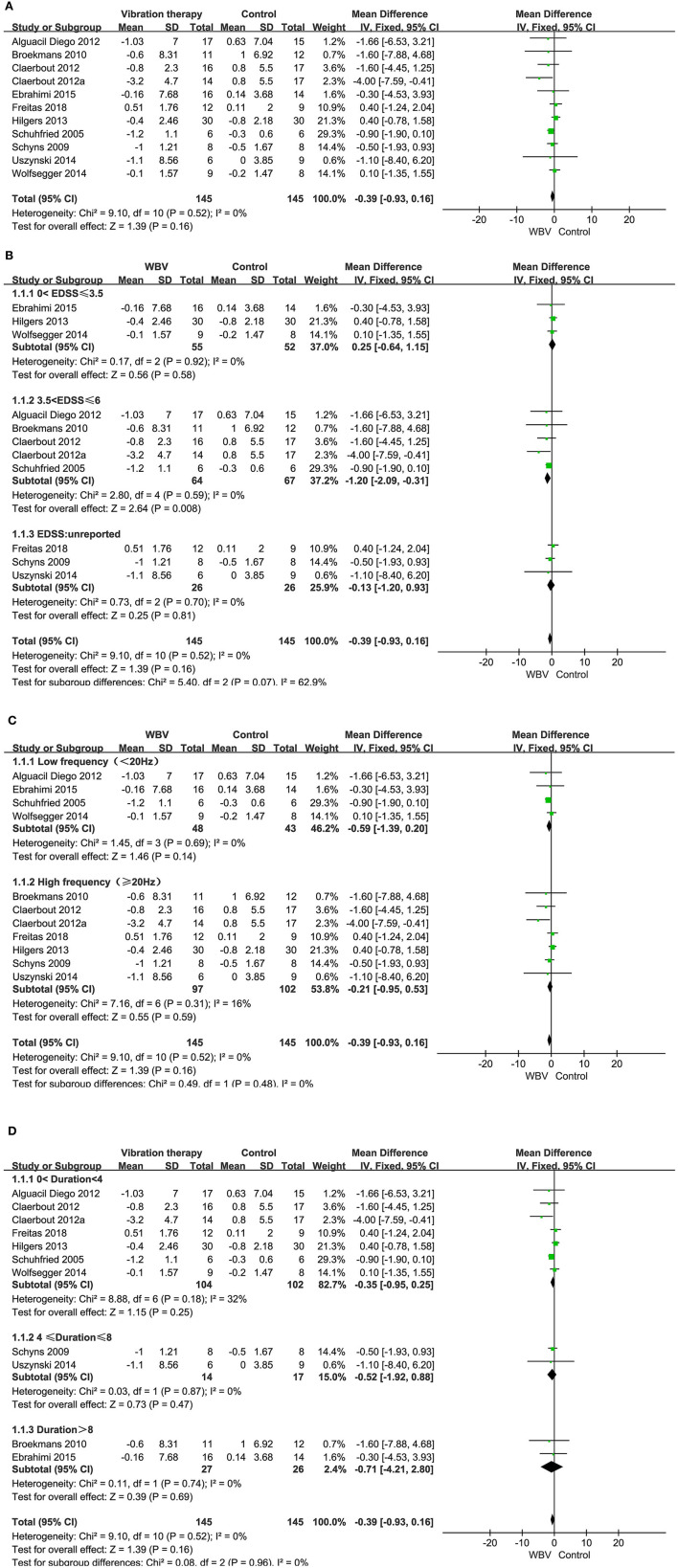
**(A)** Forest plot showing the effects of WBV on functional mobility. **(B)** Forest plot showing the effects of WBV on functional mobility in subgroups stratified according to the EDSS score. **(C)** Forest plot showing the effects of WBV on functional mobility in subgroups stratified according to the frequency. **(D)** Forest plot showing the effects of WBV on functional mobility in subgroups stratified according to the different durations of the intervention.

Ten studies with 290 patients using TUG as the measurement were included in the meta-analysis to evaluate functional mobility. Ten studies examined the effects of WBV interventions on TUGT. A fixed-effects model was used, as low heterogeneity existed (*P* = 0.52, *I*^2^ = 0%). The combined results demonstrated that WBV had no significant advantage over the control group in improving functional mobility (MD: −0.39, 95% CI: −0.93 to 0.16, *P* = 0.16) (Figure 4A).

Taking into account whether the vibration exposure parameters and degree of disability affect functional mobility, subgroup analyses were performed based on EDSS, duration of the intervention, and frequency.

Subgroup analysis based on the degree of disability: only one study did not report an EDSS score, and the rest of the studies reported an EDSS score between 0-6.5. We classified the EDSS into 0–3.5, 3.5–6, and unreported. No significant differences were observed among the subgroups (*P* = 0.99, *I*^2^ = 0%). The EDSS score (0–3.5) showed no improvement in TUG (MD: 0.25, 95% CI: −0.64 to 1.15, *P* = 0.58) (Figure 4B). However, the EDSS score (3.5–6) of MS in three articles resulted in a significant difference in improving functional mobility compared with the control group (MD: −1.20, 95% CI: −2.09 to −0.31, *P* = 0.008).

Subgroup analysis based on vibration frequency: the vibration frequency of the nine studies ranged from 2 to 45 Hz and was classified into low frequency (<20 Hz) and high frequency (≥20Hz). No significant differences were observed among the subgroups (*P* = 0.48, *I*^2^ = 0%). The subgroup analysis revealed that both the low frequency (MD: −0.59, 95% CI: −1.39–0.20, *P* = 0.14) and high frequency had no significant advantage over the control group in improving functional mobility (MD: −0.21, 95% CI: −0.95–0.53, *P* = 0.59) (Figure 4C).

Subgroup analysis based on different durations of the intervention: the intervention period of nine studies ranged from 5 days to 20 weeks. We classified the duration of the intervention into 0–4, 4–8, and >8 weeks. No significant differences were observed among the subgroups (*P* = 0.96, *I*^2^ = 0%). The subgroup analysis revealed that none of the three subgroups improved significantly in the WBV group compared to the control (MD: −0.35, 95% CI: −0.95 to 0.25, *P* = 0.25, 0 < duration < 4; MD: −0.52, 95% CI: −1.92 to 0.88, *P* = 0.47, 4 ≤ duration ≤ 8; MD: −0.71, 95% CI: −4.21 to 2.80, *P* = 0.69, duration > 8) (Figure 4D).

###### Sensitivity analysis

To assess the robustness of the results, we performed sensitivity analyses by eliminating each study and rerunning the analysis, the result of sensitivity analyses found that the combined results were stable and not affected by a single dataset (Supplementary Figure 1).

###### Balance

**Figure 5:**
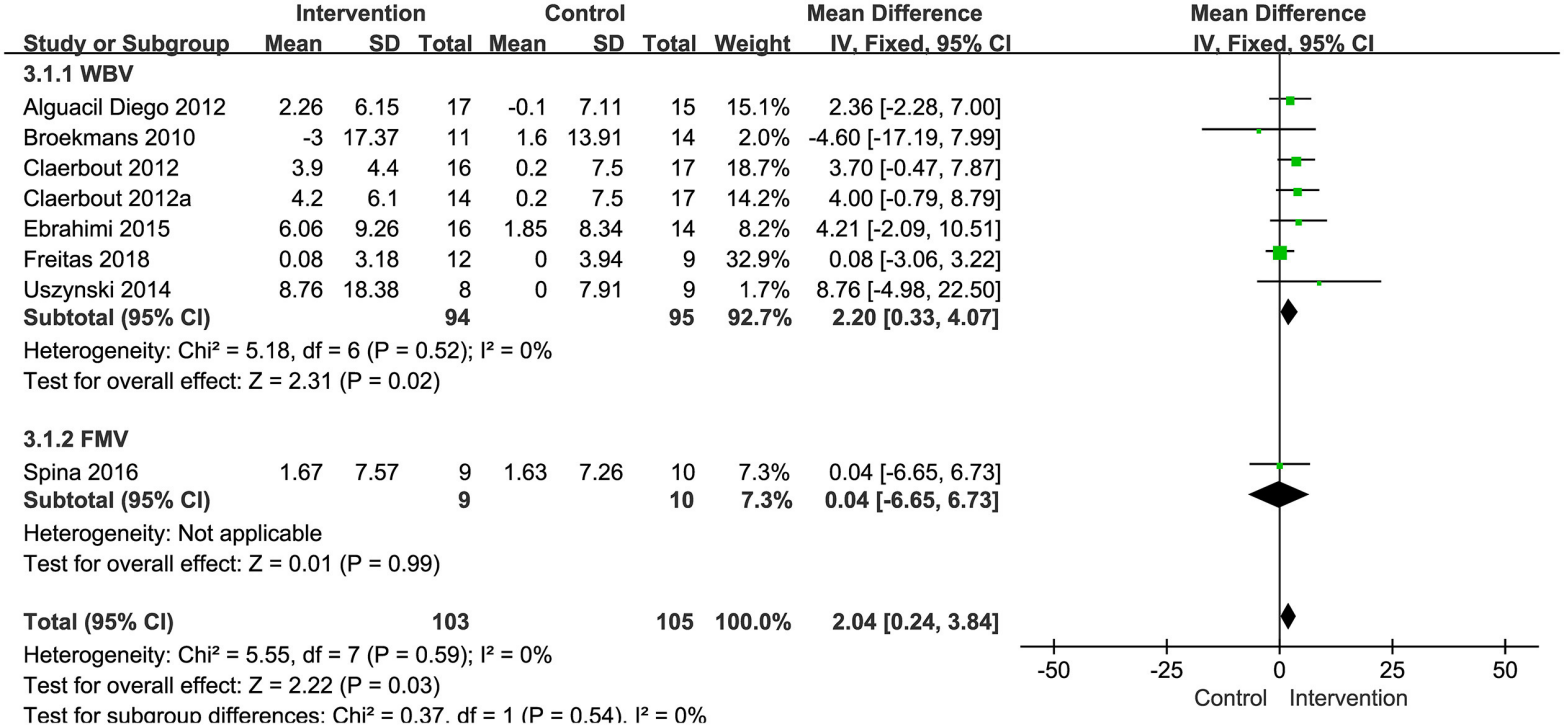
Forest plot showing the effects of vibration therapy on balance.

**Figure 6:**
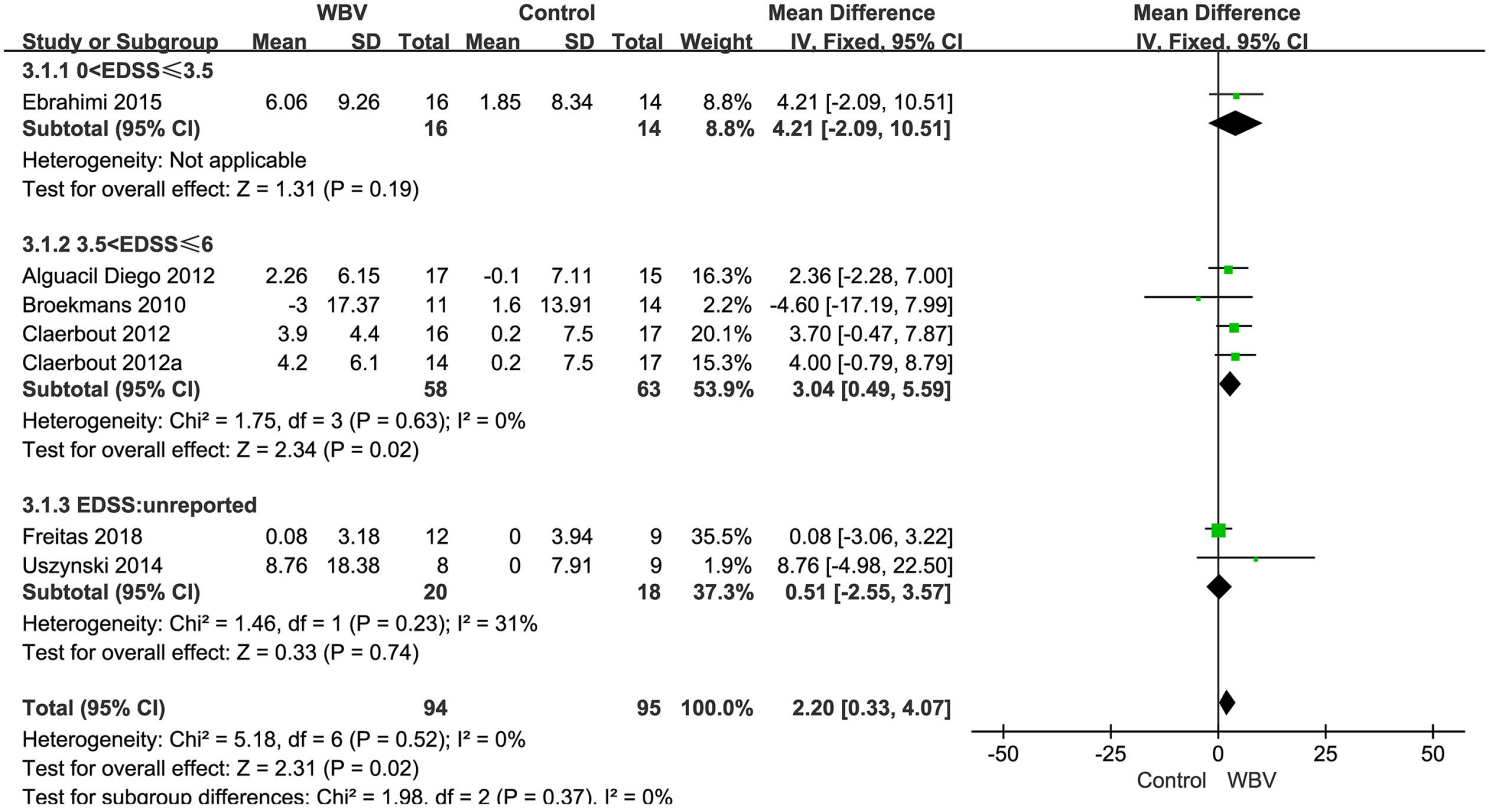
Forest plot showing the effects of WBV on balance in subgroups stratified according to the EDSS score.

Seven studies with 208 patients using BBS as the measurement were included in the meta-analysis to evaluate balance. Six studies examined the effects of WBV interventions on BBS scores, and only one study examined the effects of FV interventions. A fixed-effects model was used, as low heterogeneity existed (*P* = 0.59, I^2^ = 0%). The combined results demonstrated that these two types of vibration therapy had a significant advantage over the control group in improving balance function (MD: 2.04, 95% CI: 0.24–3.84, *P* = 0.03) (Figure 5). Six studies examining WBV showed advantages in BBS scores compared with the control group (MD: 2.20, 95% CI: 0.33–4.07, *P* = 0.02) (Figure 5). However, only one study examining FMV did not show a significant difference in improving balance function (MD: 0.04, 95% CI: −6.65 to 6.73, *P* = 0.99) (Figure 5). We also performed subgroup analyses of BBS based on the degree of disability, duration of the intervention, and frequency. No significant differences were observed among the subgroups (*P* = 0.37, *I*^2^ = 0%, EDSS; *P* = 0.80, *I*^2^ = 0%, duration; *P* = 0.62, *I*^2^ = 0%, frequency). The subgroup analysis only indicated that the WBV group was more sensitive to improving BBS scores than the control group when the EDSS scores ranged from 3.5 to 6 (MD: 3.04, 95% CI: 0.49–5.59, *P* = 0.02) (Figure 6). Other subgroup analysis results were not significant (Supplementary Figures 2, 3).

###### Walking endurance

**Figure 7:**
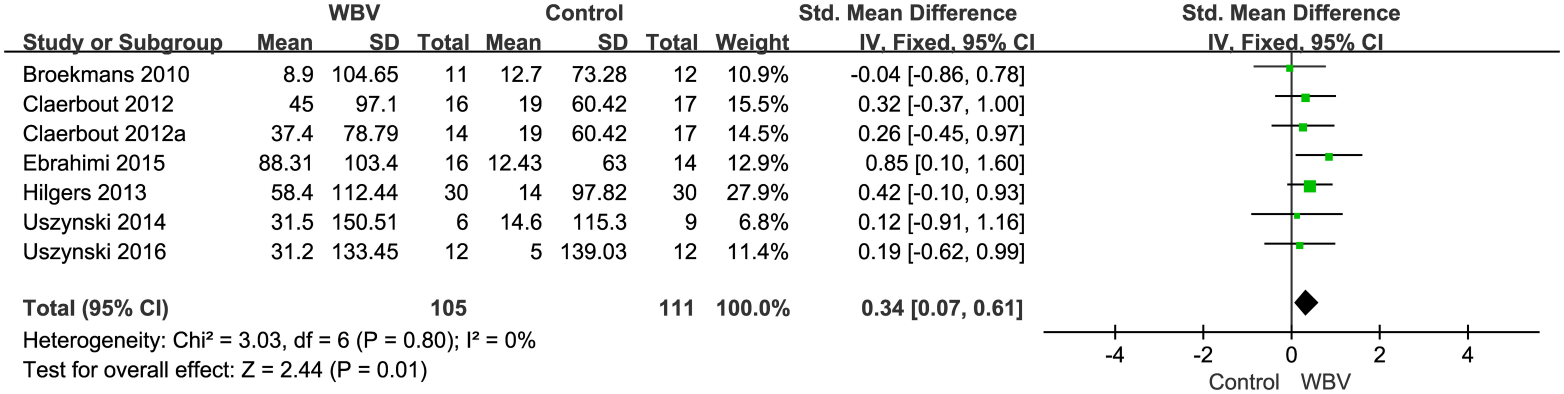
Forest plot showing the effects of WBV on walking endurance.

**Figure 8:**
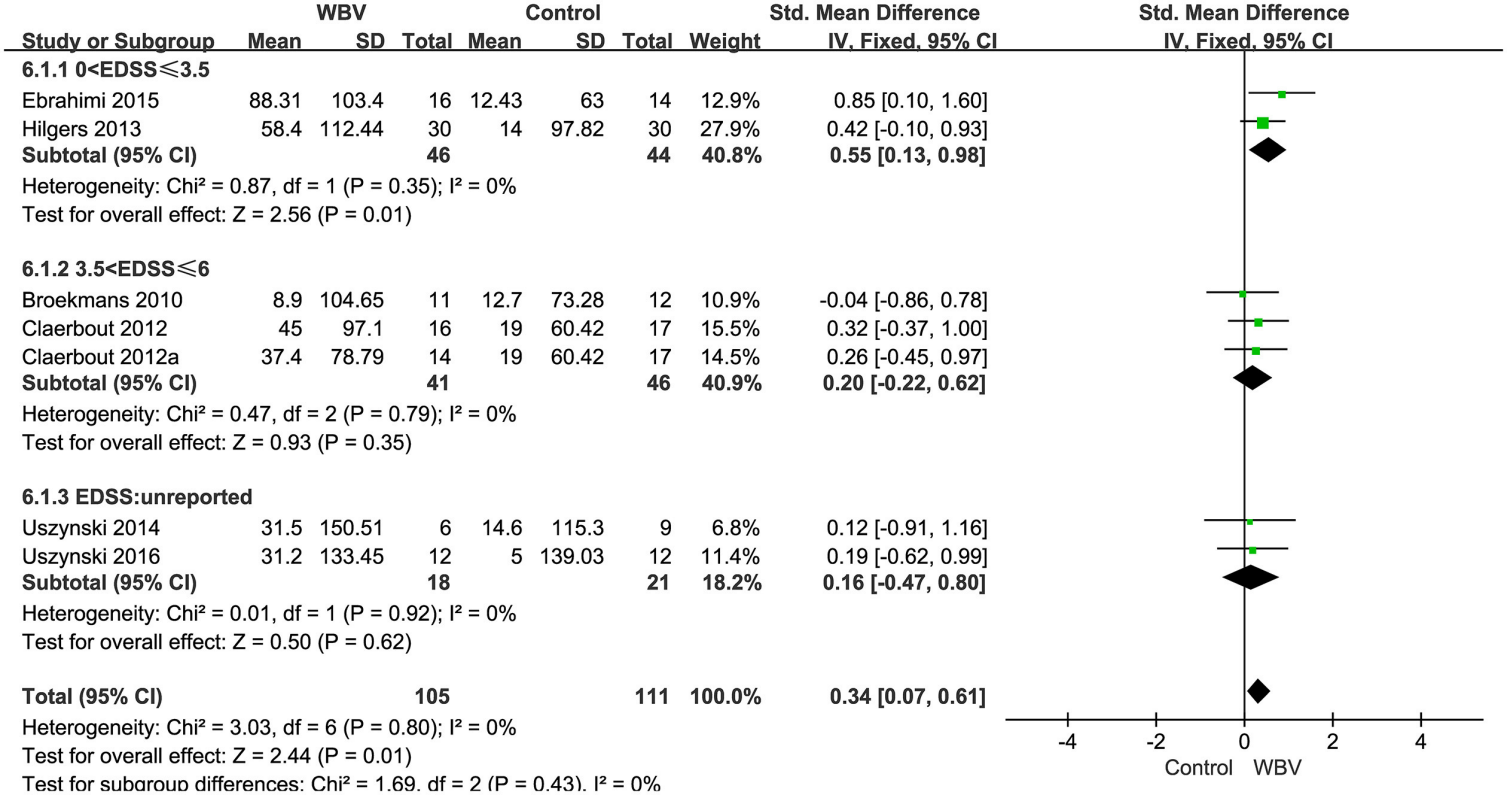
Forest plot showing the effects of WBV on walking endurance in subgroups stratified according to the EDSS score.

**Figure 9:**
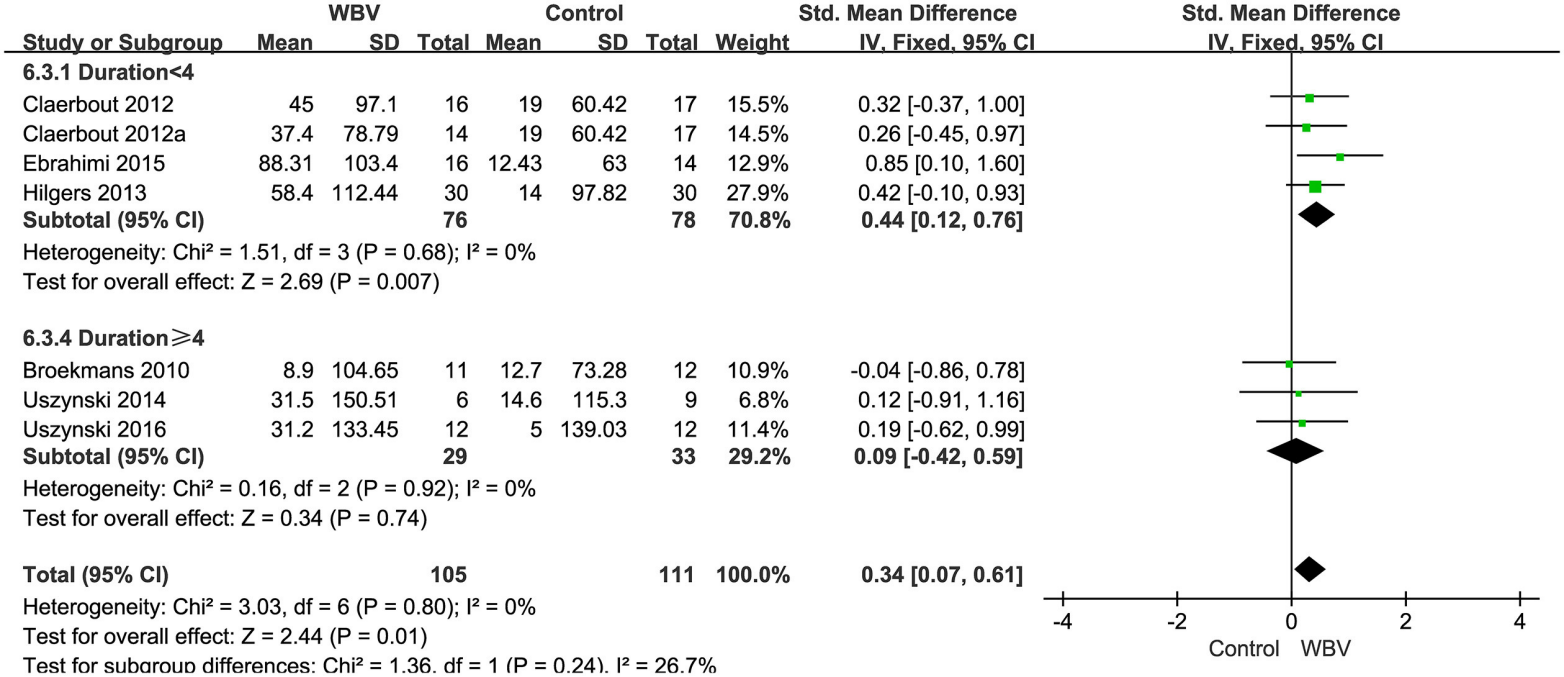
Forest plot showing the effects of WBV on walking endurance in subgroups stratified according to the different durations of the intervention.

Six studies with 216 patients used 6MWT, 2MWT, or 3MWT to assess the walking endurance. We calculated the SMD to eliminate the difference. All studies examined the effects of WBV interventions on walking endurance. A fixed-effect model was used since no heterogeneity existed (*P* = 0.8, *I*^2^ = 0%). The pooled result showed that WBV had a significant effect on improving the walking endurance with MS (SMD: 0.34, 95% CI: 0.07–0.61, *P* = 0.01) (Figure 7). We performed subgroup analyses of walking endurance based on the degree of disability (0–3.5; 3.5–6; unreported) and durations of the intervention (<4 weeks; ≥4 weeks). No significant differences were observed among the subgroups (*P* = 0.43, *I*^2^ = 0%, EDSS; *P* = 0.45, *I*^2^ = 0%, duration). The subgroup analysis indicated that the WBV group was more sensitive to improving walking endurance than the control group when the EDSS score ranged from 0 to 3.5 (SMD: 0.55, 95% CI: 0.13–0.98, *P* = 0.01) (Figure 8) and the duration of the intervention was less than 4 weeks (SMD: 0.46, 95% CI: 0.04–0.87, *P* = 0.03) (Figure 9). Other subgroup analysis results were not significant (Supplementary Figure 4).

###### Gait speed

**Figure 10:**
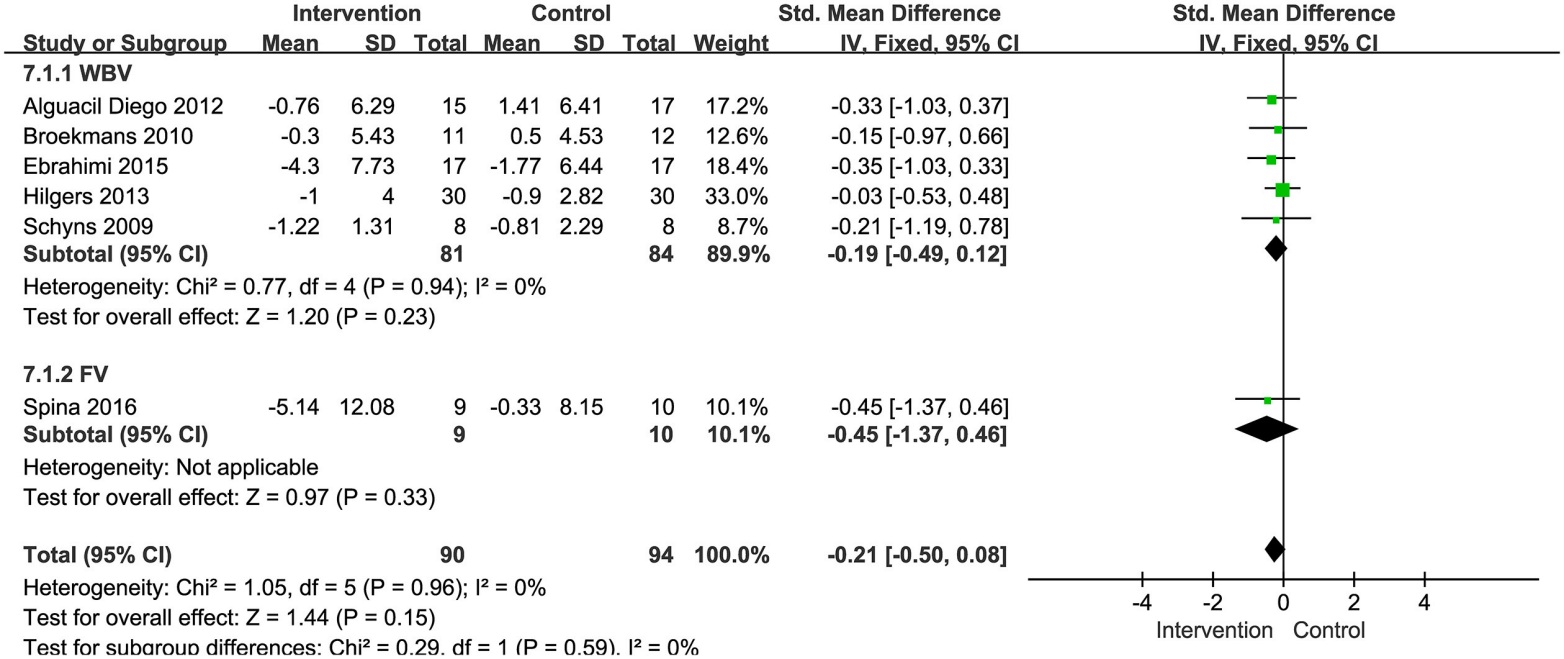
Forest plot showing the effects of vibration therapy on gait speed.

Six included RCTs with 184 patients used 10 mWT or 25FWT to assess the gait speed. We calculated the SMD to eliminate the difference. One study examined the effects of FV interventions on gait speed, other studies examined the effects of WBV interventions. A fixed-effect model was used since no heterogeneity existed (*P* = 0.96, *I*^2^ = 0%). The combined results demonstrated that these two types of vibration therapy had no significant advantage over the control group in improving gait speed (SMD: −0.21, 95% CI: −0.50 to 0.08, *P* = 0.15) (Figure 10). We performed subgroup analyses of gait speed based on the degree of disability (0–3.5; 3.5–6), frequency (<20 Hz, ≥20 Hz), and duration of the intervention (<4 weeks; ≥4 weeks). No significant differences were observed among the subgroups (*P* = 0.44, *I*^2^ = 0%, EDSS; *P* = 0.43, *I*^2^ = 0%, frequency*; P* = 0.70, *I*^2^ = 0%, duration). All subgroup analysis results were not significant (Supplementary Figures 5–7).

##### Non-motor impairments outcomes assessment

###### Fatigue

**Figure 11:**
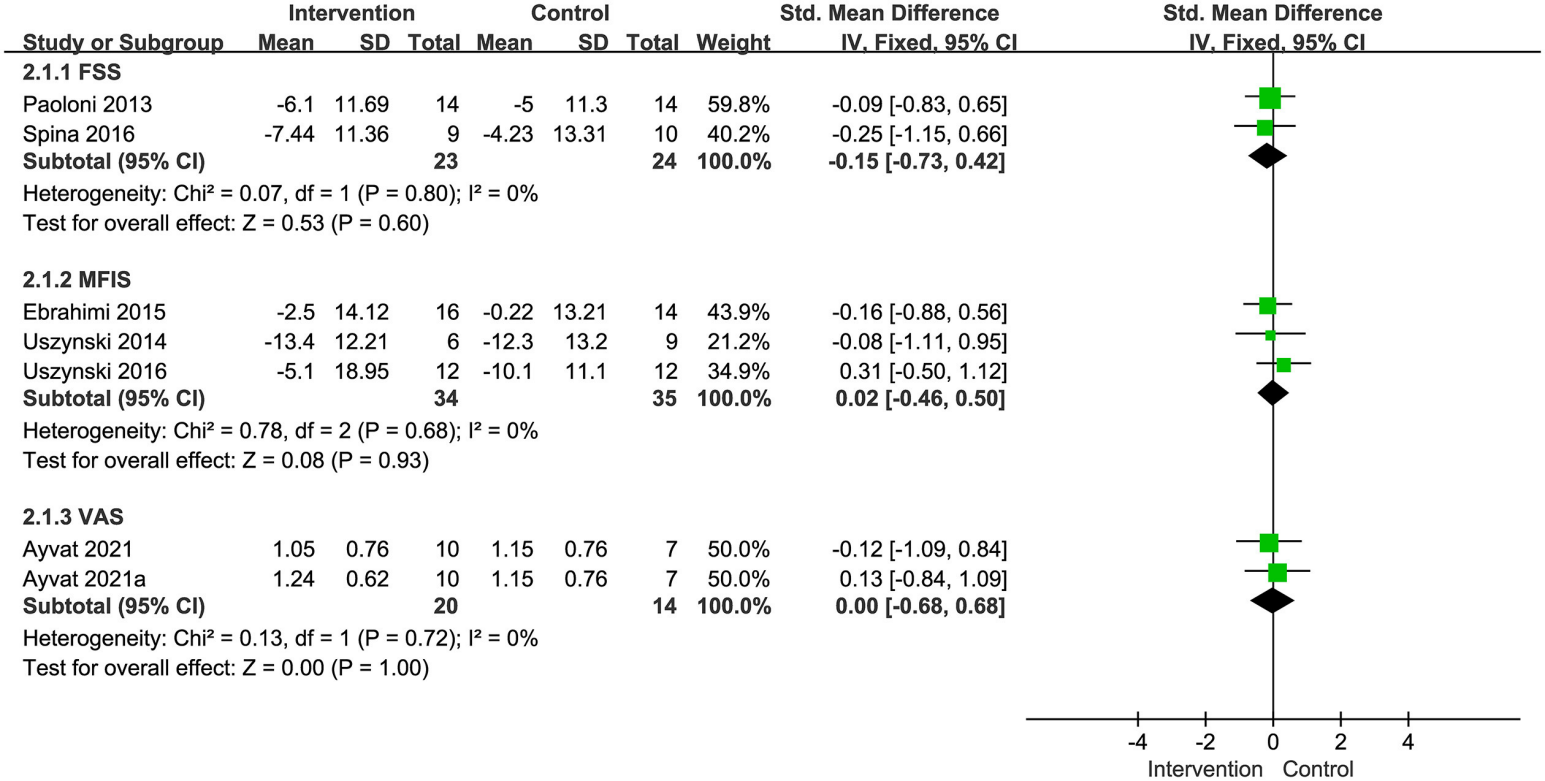
Forest plot showing the effects of vibration therapy on fatigue.

Two studies with 45 patients selected FSS as an outcome measure to assess the fatigue, and this meta-analysis found a non-significant pooled effect size (SMD: −0.15; 95% CI:−0.73–0.42, *P* = 0.60; *I*^2^ = 0%) (Figure 11). Three studies with 69 patients used the MFIS to assess the fatigue, and this meta-analysis showed a non-significant pooled effect size (SMD:0.02; 95% CI:−0.46–0.50, *P* = 0.93; *I*^2^ = 0%) (Figure 11). One study with two frequencies of intervention used VAS to assess the fatigue, and this meta-analysis showed a non-significant pooled effect size (SMD:0.00; 95% CI:−0.68-0.68, *P*=1; *I*^2^ = 0%) (Figure 11).

###### Health-related quality of life

The health-related quality of life consists of physical and mental components. One study selected MSQOL-54 and two studies used MSIS-29 as an outcome measure. The results demonstrated that vibration therapy had no significant advantage over the control group in improving physical and mental health (Supplementary Figures 8, 9).

###### Publication bias

Publication bias assessments are presented based on funnel plots and Egger's test. From the roughly symmetrical shapes of these funnel plots and Egger's test result, no significant publication bias was observed in studies evaluating functional mobility and balance (Supplementary Figures 10, 11; Supplementary Tables 1, 2).

## Discussion

Multiple sclerosis is a chronic neuroinflammatory disease, which has an early disease onset, a progressive course, and a very long duration with a median survival time of about 40 years from diagnosis (Weinshenker, 1994; Kesselring and Beer, 2005). Multiple symptoms can appear with fatigue and walking disability is reported to be among the most debilitating (Zhang et al., 2021). The natural history study found that around 50% use walking aids, 29% need a wheelchair, and 50–80% become unable to work (Weinshenker, 1994). Therefore, reducing the progression of disability is of interest.

Rehabilitation can be a beneficial treatment strategy for people with MS to ease the burden of these symptoms by managing symptoms, restoring function, optimizing the quality of life, promoting wellness, and boosting participation in activities of daily living (Kesselring and Beer, 2005; Khan and Amatya, 2017; Motl et al., 2017). This systematic review and meta-analysis sought to evaluate the relationship between vibration therapy in patients with MS. We performed a meta-analysis of 14 RCTs with 393 participants. The results demonstrate that vibration therapy has a positive effect on improving balance function and walking endurance. In addition, the subgroup analysis results showed that WBV might be more sensitive to improving functional mobility, balance, and walking endurance when the EDSS scores ranged from 3.5 to 6. We also concluded that WBV with a duration of <4 weeks potentially improved walking endurance. However, some subgroup analyses were performed only including a few articles, so the results need to be treated with caution. Certainly, We did not find any other meta-analysis that discussed the effect of EDSS, duration of the intervention, and frequency of vibration on improving motor symptoms in patients with MS. Our findings might add new insights to the current literature.

Concerning the possible mechanisms as regard to the overall results of our study. We speculate that neurogenic adaptations and post-activation potentiation mechanisms might explain the balance function and walking endurance after vibration therapy (Bazett-Jones et al., 2008; Rittweger, 2010). Vibration therapy improves balance through activating the Ia and II afferents of muscle groups and inducing sensory stimulation of foot-sole afferents, increasing the sensitivity of muscle mechanoreceptors (i.e. Golgi tendon organs and muscle spindle) and changing joint stiffness (Kavounoudias et al., 1999; Fontana et al., 2005; Siu et al., 2010; Ritzmann et al., 2014; Abdel-Aal et al., 2021).

In addition, the improvement in walking endurance after vibration is attributed to enhanced capacity for transporting and utilizing oxygen in the muscle (e.g. increased blood flow to the active skeletal muscle) (Bogaerts et al., 2009; Games et al., 2015; Betik et al., 2021), improvements in skeletal muscle function and morphology (e.g. hypertrophy, increase in capillary density) and increase VO_2peak_ (Aoyama et al., 2019).

Reduced mobility is probably the commonest impairment compromising daily living activities of subjects affected by MS with moderate to severe walking disability (Gijbels et al., 2010; Gianni et al., 2014). In our study, the outcome of the TUG test measurements is described as functional mobility-a formulation of combining balance, gait, and mobility, which is an excellent tool used to assess mobility capabilities in MS, monitor disease progression, and identify potential MS fallers (Kalron et al., 2017). Our results demonstrated that vibration therapy was not a significant improvement for functional mobility similar to other studies (Schyns et al., 2009; Broekmans et al., 2010; Uszynski et al., 2014; Wolfsegger et al., 2014; Ebrahimi et al., 2015; Freitas et al., 2018; Alam et al., 2020). The results were persistent and stable when sensitivity analyses were conducted. On the other hand, the whole-body vibration exercise also did not improve functional mobility in a randomized, multi-center, parallel, clinical study in older people (Sitja-Rabert et al., 2015), which is consistent with our results. However, from an intervention perspective, these findings may be related to significant differences between patients, interventions (e.g. frequency, duration, equipment, etc.), and control (e.g. exercise alone and placebo). Thus, we did subgroup analysis based on EDSS scores, duration of the intervention, and frequency. The results showed that the effect of WBV was superior to the conventional therapy paradigm with the EDSS score ranging 3.5 from to 6, but the duration and frequency did not show an ideal effect on functional mobility. One hypothesis may be that EDSS may affect the effectiveness of therapy (Haselkorn et al., 2015). The mild disability (EDSS ≤ 3.5) may already have been performing at their individual maximal physical activity level with less potential to improve (Claerbout et al., 2012). Meanwhile, the transfers on/off the floor and into/out of chairs are increasingly challenging with moderate to severe disability (EDSS, range, 3.5-6) (Kalb et al., 2020), which have great potential for improvement.

Balance impairment is one of the most disabling symptoms in people with MS that affects about 50–80% of patients during the disease (Molhemi et al., 2021). These impairments have recently been proposed as a key mechanism of frequent falls in MS with more than 50% of MS reporting one fall or more over a 3 to 12-month period (Mohamed Suhaimy et al., 2020). In our study, we used BBS to evaluate the balance with MS. The BBS has been confirmed, test-retest and inter-rater reliability were excellent in MS (Cattaneo et al., 2007), and there is strong first-level evidence to support its use to assess changes in static and dynamic sitting balance (Moore et al., 2018; Mehta et al., 2019). Many studies have shown that vibration therapy can improve the balance function of MS (Uszynski et al., 2014; Ebrahimi et al., 2015). A meta-analysis of four RCT studies by Alam et al. (2020) showed significant improvement with WBV intervention to improve balance, which is consistent with the findings of our study. Another meta-analysis of the effects of WBV did not significantly raise the BBS score for patients with MS, but the included studies of that review are not rigorous and the sample size is small, Our study has better homogeneity and stronger conclusions. In addition, the results demonstrated no significant changes in BBS (Broekmans et al., 2010; Alguacil Diego et al., 2012; Freitas et al., 2018), and found a significant improvement within the WBV group only (Claerbout et al., 2012; Spina et al., 2016). This may be related to the higher baseline levels of BBS in these studies and the limited number of studies. Our subgroup analysis showed that the effect of WBV was more sensitive to improving balance function with the EDSS scores ranging from 3.5 to 6, similar to the functional mobility. In addition to the reasons explained above, the possible reason is that BBS captures mostly static balance and transfers without considering other facets such as dynamic balance skills that are important in situations nearer to activities of daily living (Gervasoni et al., 2017).

Walking endurance compromise is a common, life-altering feature of MS (Motl and Learmonth, 2014). The 6MWT and 2MWT have been the most commonly applied performance-based measure of endurance walking capacity in MS (Motl et al., 2012; Learmonth et al., 2013; Carpinella et al., 2021). They highlight motor fatigue resulting from extended task execution, thus, effectively assessing the physical efforts and level of autonomy of MS (Moore et al., 2018). It is important to note that the one of included studies used 3MWT to assess walking endurance (Claerbout et al., 2012). The reason was that the patients were unable to complete 6MWT due to fatigue and the walking endurance data can only be obtained by reducing walking time. However, the validity and reliability of the 3 min walk for MS remain unconfirmed to date. Considering the completeness of the data, this study was also included in our analysis. As revealed by our meta-analysis, overall heterogeneity did not change after adding this study (Claerbout et al., 2012). Previous studies have provided some evidence that WBV is beneficial for improving walking endurance in individuals with MS (Hilgers et al., 2013; Ebrahimi et al., 2015; Alam et al., 2020). Our analysis outcomes showed that WBV had a significant effect on improving the walking endurance with MS. In contrast, other studies (Broekmans et al., 2010; Uszynski et al., 2014, 2016) have not conclusively shown that WBV provides a significant advantage over the control group among MS populations due to the limited number of studies. However, these studies showed WBV improved significantly before and after treatment with values equal or greater than 21.6 m which can be considered clinically meaningful in MS (Baert et al., 2014). Our subgroup analysis showed that the effect of WBV was more sensitive to improved walking endurance when the EDSS scores ranged from 0 to 3.5 and durations of the intervention was less than 4 weeks similar to Hilgers et al. (2013). The study indicated the walking distance was found to be inversely related to the EDSS scores. MS is classified as having a mild disability when the EDSS scores <3.5, and its overall function is good, while MS requires assistive devices when the EDSS scores above 3.5 and may be accompanied by a reduction of muscle strength and cardiorespiratory endurance (Chetta et al., 2004). The effect of WBV alone on improving walking endurance may be more pronounced at lower disability levels. However, the duration (>4 weeks) did not show an ideal effect on walking endurance, it might be due to the patients reaching a plateau where no significant clinical benefit was observed. In summary, the optimal WBV training parameters for improving walking endurance was recommended to be per week with a duration of less than 4 weeks for MS of EDSS scores less than 3.5.

A deterioration in gait speed occurs very early following diagnosis of MS when people report no or minimal limitations in function (Martin et al., 2006; Cattaneo et al., 2022). The 10MWT and 25FWT provide a performance-based measure of walking dysfunction based on walking speed over a short distance, which are the common objective measure for characterizing walking dysfunction among persons with MS (Feys et al., 2014; Amatachaya et al., 2020; Sikes et al., 2020; Kalinowski et al., 2022). The studies found that the 10MWT and 25FWT demonstrated more significant changes in those with greater disability of MS with a very high correlation (Feys et al., 2014; Williams et al., 2016). Two of included studies used 25FWT (Broekmans et al., 2010; Spina et al., 2016), and four of included studies used 10MWT (Schyns et al., 2009; Alguacil Diego et al., 2012; Hilgers et al., 2013; Ebrahimi et al., 2015). Many studies have shown that vibration therapy is not beneficial for improving gait speed in individuals with MS (Schyns et al., 2009; Broekmans et al., 2010; Alguacil Diego et al., 2012; Hilgers et al., 2013; Ebrahimi et al., 2015; Spina et al., 2016). A meta-analysis of 3 studies reported WBV was no significant improvement in 10MWT (Alam et al., 2020). Another meta-analysis of 3 studies revealed that the WBV was not significantly associated with the walking speed (Kang et al., 2016). Our meta-analysis showed that vibration therapy did not significantly improve gait speed, consistent with the above studies. Of course, our subgroup analysis results were also negative. Gait speed is a valid, reliable, sensitive measure appropriate for assessing and monitoring functional status and overall health in a wide range of populations (Middleton et al., 2015). The improvement of gait speed is affected by many factors, and the dysfunctional features of MS are multidimensional (e.g., balance and mobility impairments, weakness, reduced cardiovascular fitness, ataxia, fatigue, pain, cognitive deficits, depression, etc.) (Feinstein et al., 2015). Vibration therapy alone may not be effective, which is also found in stroke patients (Brogardh et al., 2012; Moggio et al., 2021).

Additional studies have strong evidence that WBV can be increased to improve gait speed in older adults by assessing 10MWT (Fischer et al., 2019; Wadsworth and Lark, 2020). The above evidence shows that WBV is affected by the type of disease in improving gait speed. In addition, numerous factors can affect the results of the program (e.g., the duration of the intervention, the frequency or volume of the sessions; the type, frequency, and amplitude of the vibrations, and the exercises performed on the platform) (Wadsworth and Lark, 2020). Therefore, a standardized vibration therapy regimen needs to be developed.

Fatigue is the most common and debilitating symptom of MS and has a significant impact on virtually all aspects of an individual's daily functioning (Latimer-Cheung et al., 2013). It typically is measured through self-report questionnaires (Krupp, 2004). Measures included in our study are the FSS, MFIS, and VAS. A few studies have reported that WBV is not a significant reduction in MFSI (Uszynski et al., 2014, 2016; Ebrahimi et al., 2015). The results reported by Paoloni et al. (2013) and Spina et al. (2016) found FMV was significantly improved in FSS, whereas there was no difference between the FMV and control groups. Only one study used VAS to evaluate the fatigue, which increased in all groups. Our meta-analysis showed that vibration did not result in a significant improvement in fatigue compared with the control group. One possible reason is that patients in included studies had different baseline fatigue levels, which may have affected the results. Furthermore, the self-report questionnaires are entirely subjective and confounded by other symptoms of MS (Schwid et al., 2002). Considering the importance of improving fatigue in MS patients, recent studies have suggested that a multimodal approach should be used to address fatigue in persons with MS, combining psychological and physical aspects (Carter et al., 2014).

MS patients report lower HRQoL as compared to general and other chronic disease populations (Berrigan et al., 2016). Restricted walking prevents MS patients from participating in family and social activities and is a major determinant of overall impairment (Schwid et al., 1997). Currently, few studies have examined the impact of vibration training on the health-related quality of life in MS patients. The studies we included mainly used the MSQL-54 and the MSIS-29 to assess the health-related quality of life. However, there was insufficient evidence of added benefit from the whole body vibration. Both intervention programs facilitate the patient's socialization, which in itself may have contributed to some of the beneficial effects (Ebrahimi et al., 2015). This finding corroborates previous results suggesting that exercise, regardless of the type, has a strong positive effect on the physical and psychological impact of multiple sclerosis (Heine et al., 2015). The quality of life is affected by multiple factors, including interpersonal relations, life environment, psychological and physical state, as well as individual life satisfaction (Gil-Gonzalez et al., 2020). Therefore, recent studies have suggested that a multimodal approach should be used to address health-related quality of life in persons with MS, combining psychological and physical aspects (Carter et al., 2014).

### Strengths and limitations

Our systematic review comprehensively explored the effects of Vibration therapy as a treatment on patients with MS. Although there are currently systematic reviews and meta-analyses of the effects of whole-body vibration therapy on motor function in patients with MS, our systematic review and meta-analysis may be the first to explore the effects of vibration therapy on non-motor symptoms, and to perform subgroup analyses on the motor function to illustrate the effects of disability, vibration frequency, and duration of intervention. However, there are several limitations to this review. First, 14 studies were included, but the overall sample size was small; Second, the strength and accuracy of the conclusions of NMS are limited by the small number of eligible studies available; Third, the subgroup analyses were performed only included a few articles, which might increase the deviation of results; Fourth, the studies reported were inconsistent about vibration exposure. Finally, the long-term effectiveness of vibration therapy should be investigated, because it holds significant value in clinical practice.

## Conclusion

In conclusion, the present systematic review and meta-analysis suggested that vibration therapy may be more beneficial to improve balance function and walking endurance. Nevertheless, the degree of disability and duration of intervention may affect outcomes. However, there is insufficient evidence to demonstrate that vibration therapy is effective in the treatment of functional mobility, gait speed, fatigue, and quality of life of patients with MS. Further multi-center research with larger sample sizes is needed. Meanwhile, future research should focus on determining the vibration parameters that are most beneficial to the functional recovery of patients with MS.

## Data Availability Statement

The original contributions presented in the study are included in the article/Supplementary material, further inquiries can be directed to the corresponding author/s.

## Author contributions

YZ and PX put forward this review and designed the protocol and performed the statistical analysis. PX, YD, JC, and WD conducted the literature search and extracted and interpreted data. YZ wrote the first draft of the manuscript. MW and CN provided suggestions on writing and revised the article. All authors read and approved the final manuscript.

## Funding

This work was supported by Anhui Province Twelfth Five-Year Clinical Medicine Key Specialty (Grant No. ZD123_0013), Key Technologies R&D Program of Anhui Province (Grant No. 202004h07020017), and the Fundamental Research Funds for the Central Universities (Grant No. WK9110000134).

## Conflict of interest

The authors declare that the research was conducted in the absence of any commercial or financial relationships that could be construed as a potential conflict of interest.

## Publisher's note

All claims expressed in this article are solely those of the authors and do not necessarily represent those of their affiliated organizations, or those of the publisher, the editors and the reviewers. Any product that may be evaluated in this article, or claim that may be made by its manufacturer, is not guaranteed or endorsed by the publisher.
