# Supplementary material for: Effects of vibration training on motor and non-motor symptoms for patients with multiple sclerosis: A systematic review and meta-analysis
Source: Front Aging Neurosci. 2022 Aug 5;14:960328. doi: 10.3389/fnagi.2022.960328 (PMC9415382; doi:10.3389/fnagi.2022.960328)
Supplement: Supplementary file 1 [file Data_Sheet_1.ZIP › Supplementary Material/Supplementary_Appendix.pdf]

Supplementary Material

Appendix 1. Search strategy for each database

Pubmed search strategy

((((((((whole-body vibration[Title/Abstract]) OR (WBV[Title/Abstract])) OR (vibration[Title/Abstract])) OR (focal vibration[Title/Abstract])) OR (focal vibration therapy[Title/Abstract])) OR (focal muscle vibration[Title/Abstract])) OR (localized vibration[Title/Abstract])) OR (FVT[Title/Abstract])) AND ((multiple sclerosis[MeSH Terms]) OR (multiple sclerosis[Title/Abstract]))

Embase search strategy

#1 'focal vibration':ab,ti OR 'focal vibration therapy':ab,ti OR 'focal muscle vibration':ab,ti OR 'localized vibration':ab,ti OR 'fvt':ab,ti OR 'fmv':ab,ti

#2 'whole body vibration':ab,ti OR 'wbv':ab,ti

#3 'vibration':ab,ti

#4 #1 or #2 or #3

#5 'multiple sclerosis' OR 'multiple sclerosis'/exp

#6 'clinical trial'/exp OR 'clinical trial' OR 'controlled trial'/exp OR 'controlled trial' OR 'randomized controlled trial'/exp OR 'randomized controlled trial' OR 'random*'

#7 #4 and #5 and #6

Cochrane library search strategy

ID Search

#1 (whole-body vibration):ti,ab,kw OR (WBV):ti,ab,kw

#2 (focal vibration):ti,ab,kw OR (focal vibration therapy):ti,ab,kw OR (focal muscle vibration):ti,ab,kw OR (localized vibration):ti,ab,kw OR (FVT):ti,ab,kw OR (FMV):ti,ab,kw

#3 (vibration):ti,ab,kw

#4 MeSH descriptor: [Multiple Sclerosis] explode all trees

#5 (multiple sclerosis):ti,ab,kw

#6 #1 or #2 or #3

#7 #4 or #5

#8 #6 and #7

Web of science search strategy

#1 (((((((TS=(whole-body vibration)) OR TS=(vibration)) OR TS=(focal vibration)) OR TS=(focal vibration therapy)) OR TS=(focal muscle vibration)) OR TS=(localized vibration)) OR TS=(FVT)) OR TS=(FMV)

#2 (TS=(Multiple sclerosis)) OR TS=(MS)

#3 (((TS=(trial)) OR TS=(controlled trial)) OR TS=(random)) OR TS=(randomized controlled trial)

#4 #1 and #2 and #3

Scopus search strategy

(TITLE-ABS-KEY (whole-body AND vibration) OR TITLE-ABS-KEY (vibration) OR TITLE-ABS-KEY (focal AND vibration) OR TITLE-ABS-KEY (focal AND vibration AND therapy) OR TITLE-ABS-KEY (focal AND muscle AND vibration) OR TITLE-ABS-KEY (localized AND vibration) OR TITLE-ABS-KEY (wbv) OR TITLE-ABS-KEY (fvt) OR TITLE-ABS-KEY (fmv)) AND (TITLE-ABS-KEY (multiple AND sclerosis))

CNKI search strategy

(SU='振动训练' OR SU='全身振动训练' OR SU='局灶性振动训练') AND (SU='多发性硬化症') AND (SU='对照研究' OR ‘随机对照研究 OR ‘临床研究’)
