# Supplementary material for: Effects of vibration training on motor and non-motor symptoms for patients with multiple sclerosis: A systematic review and meta-analysis
Source: Front Aging Neurosci. 2022 Aug 5;14:960328. doi: 10.3389/fnagi.2022.960328 (PMC9415382; doi:10.3389/fnagi.2022.960328)
Supplement: Supplementary file 1 [file Data_Sheet_1.ZIP › Supplementary Material/Supplementary_Figure.pdf]

frontiers

Supplementary Material

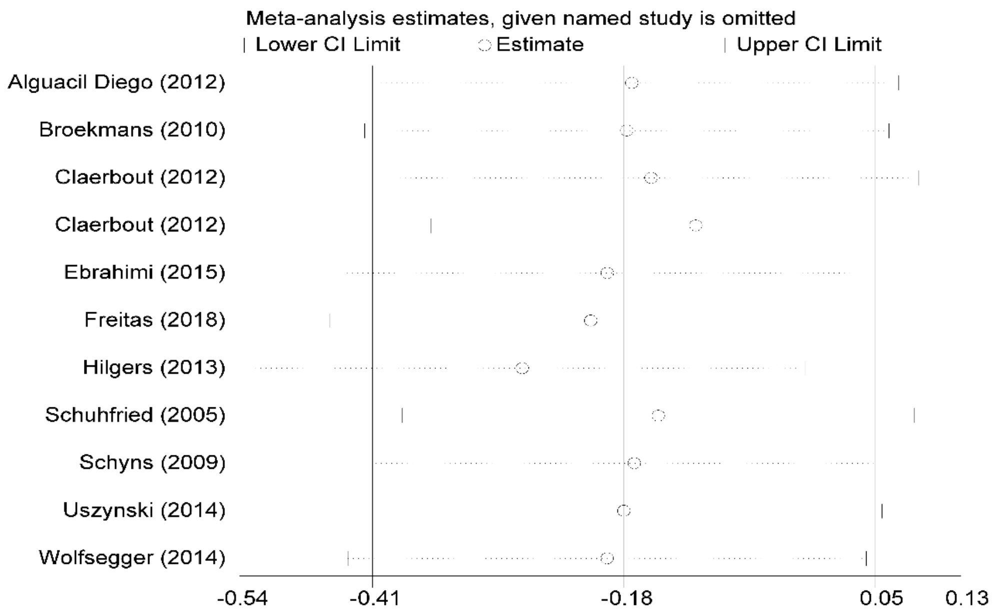

Supplementary Figure 1. The sensitivity analysis of TUG.

Study or Subgroup	WBV Mean	WBV SD	WBV Total	Control Mean	Control SD	Control Total	Weight	Mean Difference IV, Fixed, 95% CI
**3.2.1 0<Duration<4**								
Alguacil Diego 2012	2.26	6.15	17	-0.1	7.11	15	16.3%	2.36 [-2.28, 7.00]
Claerbout 2012	3.9	4.4	16	0.2	7.5	17	20.1%	3.70 [-0.47, 7.87]
Claerbout 2012a	4.2	6.1	14	0.2	7.5	17	15.3%	4.00 [-0.79, 8.79]
Freitas 2018	0.08	3.18	12	0	3.94	9	35.5%	0.08 [-3.06, 3.22]
**Subtotal (95% CI)**			**59**			**58**	**87.1%**	**2.03 [0.03, 4.03]**
Heterogeneity: Chi² = 2.77, df = 3 (P = 0.43); I² = 0%								
Test for overall effect: Z = 1.99 (P = 0.05)								
**3.2.2 4≤Duration≤8**								
Ebrahimi 2015	6.06	9.26	16	1.85	8.34	14	8.8%	4.21 [-2.09, 10.51]
**Subtotal (95% CI)**			**16**			**14**	**8.8%**	**4.21 [-2.09, 10.51]**
Heterogeneity: Not applicable								
Test for overall effect: Z = 1.31 (P = 0.19)								
**3.2.3 Duration>8**								
Broekmans 2010	-3	17.37	11	1.6	13.91	14	2.2%	-4.60 [-17.19, 7.99]
Uszynski 2014	8.76	18.38	8	0	7.91	9	1.9%	8.76 [-4.98, 22.50]
**Subtotal (95% CI)**			**19**			**23**	**4.1%**	**1.49 [-7.79, 10.78]**
Heterogeneity: Chi² = 1.97, df = 1 (P = 0.16); I² = 49%								
Test for overall effect: Z = 0.32 (P = 0.75)								
**Total (95% CI)**			**94**			**95**	**100.0%**	**2.20 [0.33, 4.07]**
Heterogeneity: Chi² = 5.18, df = 6 (P = 0.52); I² = 0%								
Test for overall effect: Z = 2.31 (P = 0.02)								
Test for subgroup differences: Chi² = 0.44, df = 2 (P = 0.80), I² = 0%								

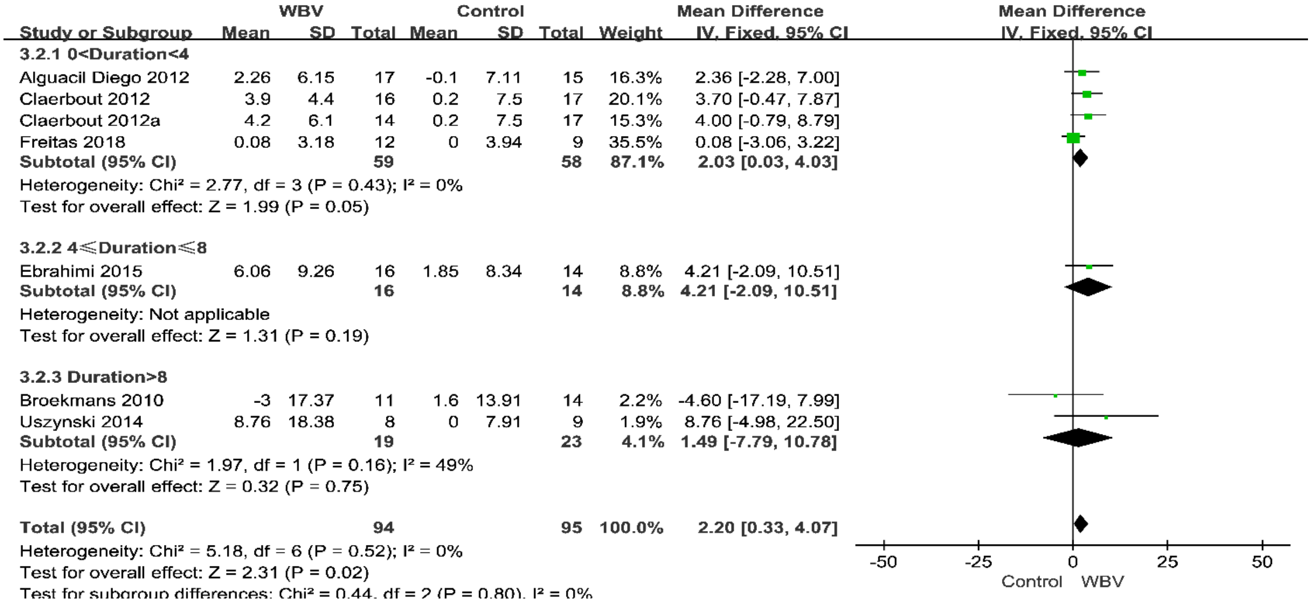

Supplementary Figure 2. The subgroup analysis results of BBS based on duration.

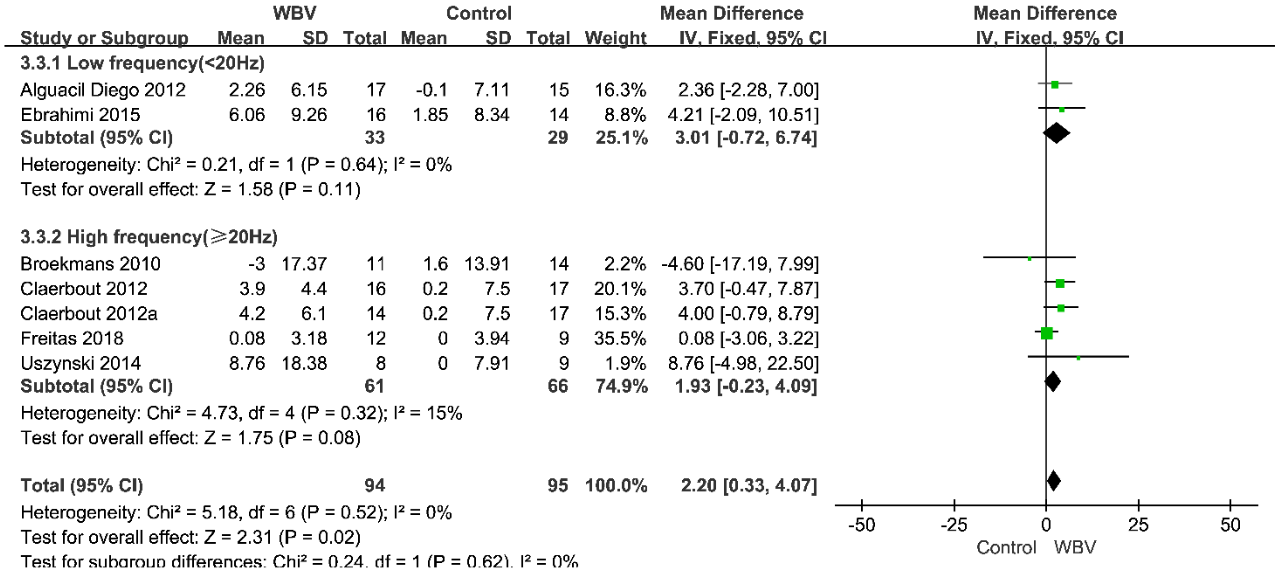

Supplementary Figure 3. The subgroup analysis results of BBS based on frequency.

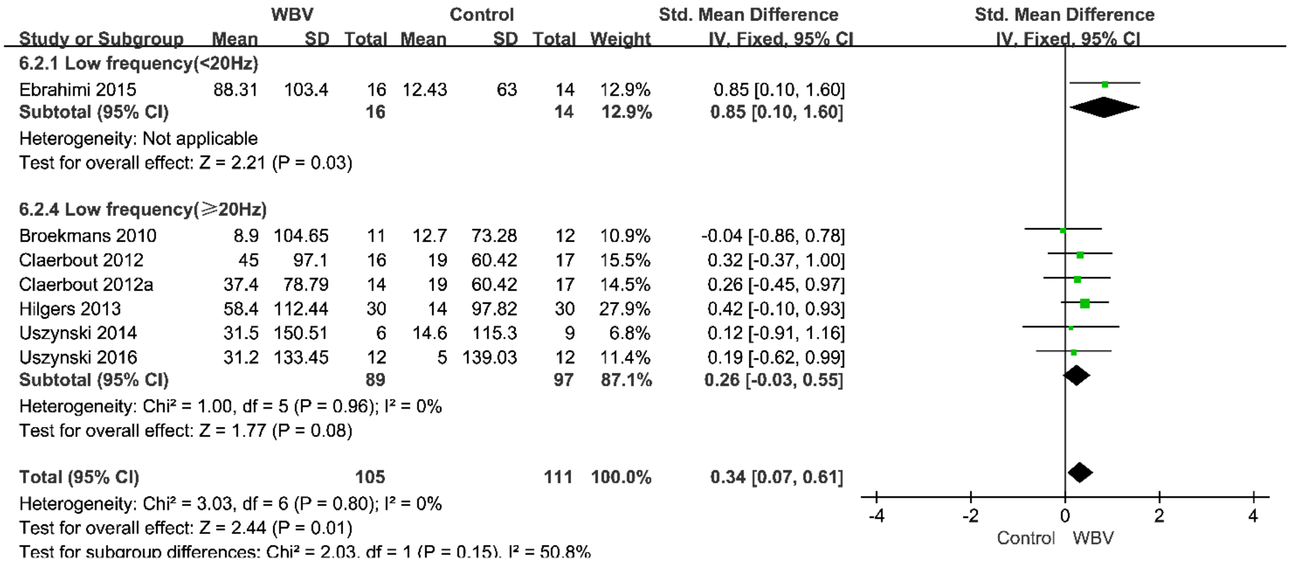

Supplementary Figure 4. The subgroup analysis results of walking endurance based on frequency.

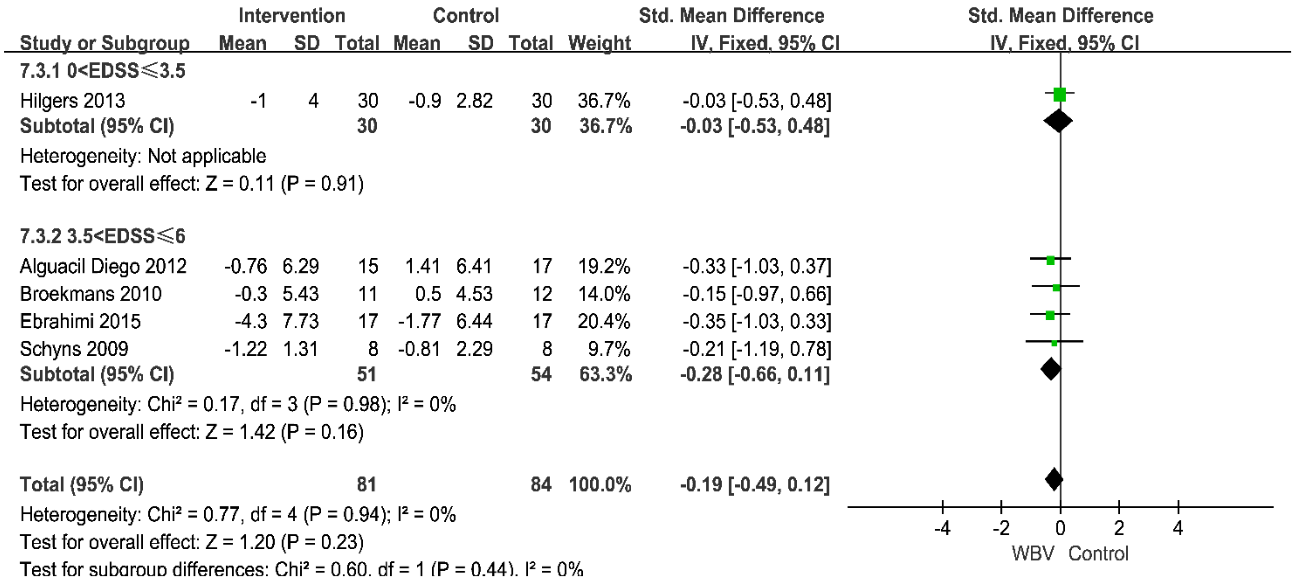

Supplementary Figure 5. The subgroup analysis results of gait speed based on EDSS.

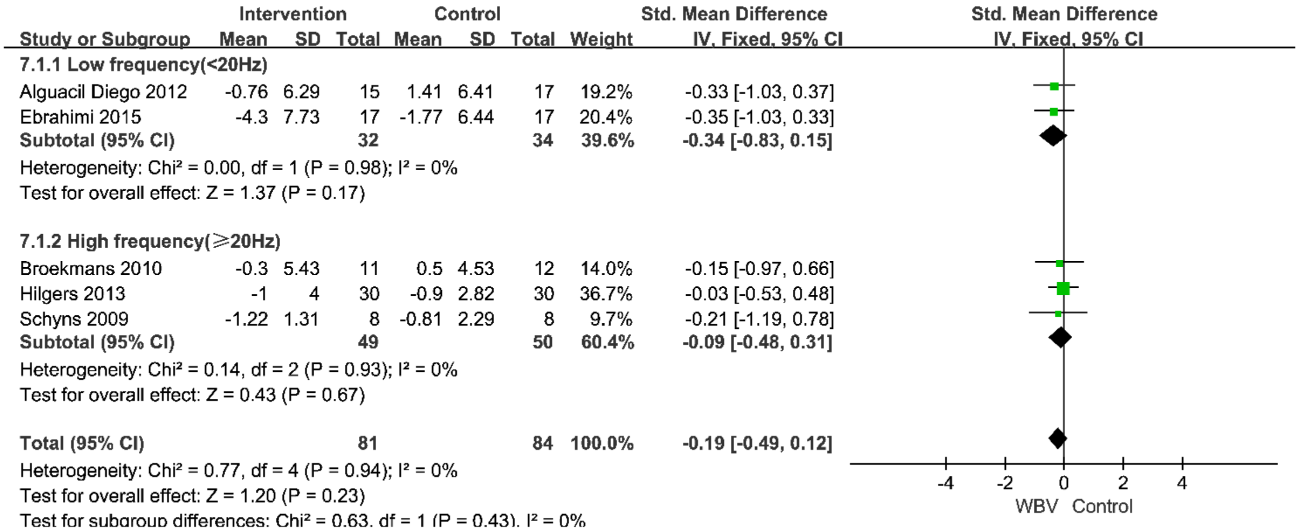

Supplementary Figure 6. The subgroup analysis results of gait speed based on frequency.

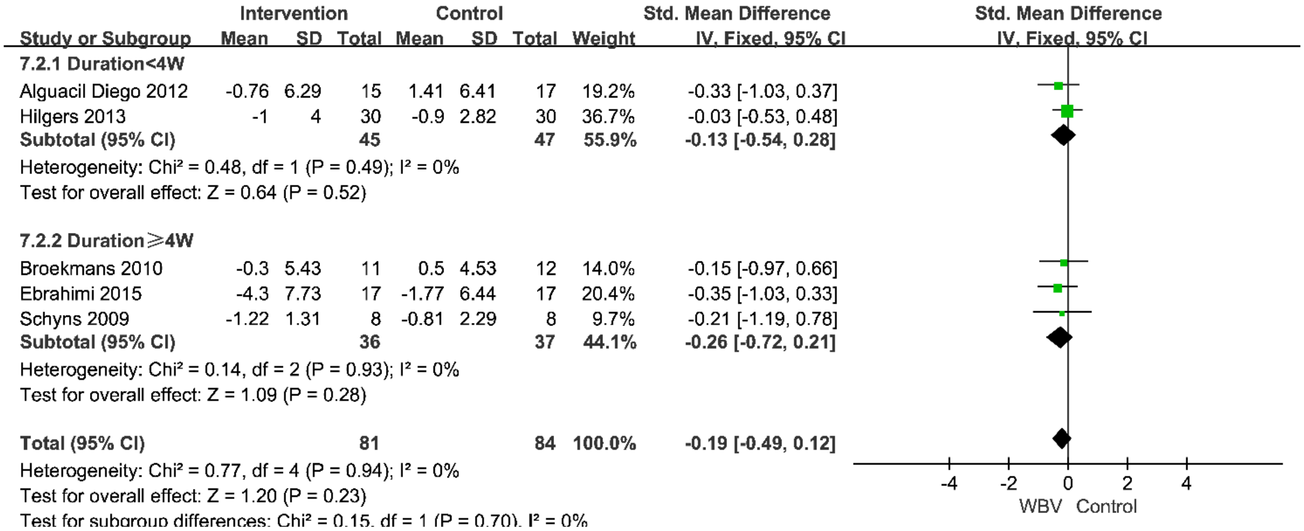

Supplementary Figure 7. The subgroup analysis results of gait speed based on duration.

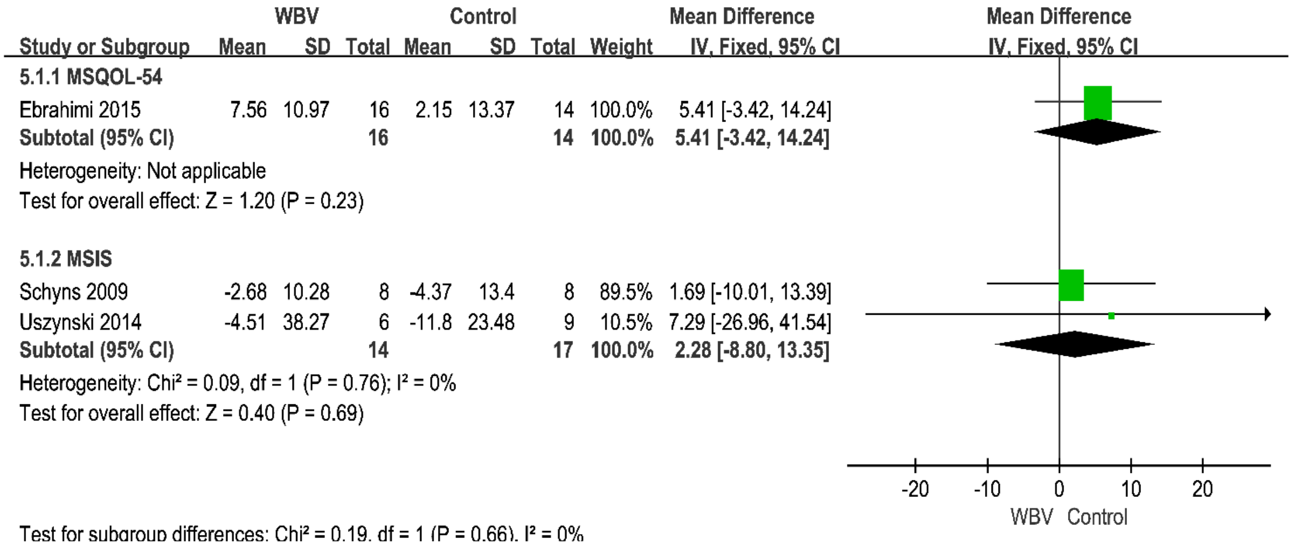

Supplementary Figure 8. The forest plot of health-related quality of life (physical healthy).

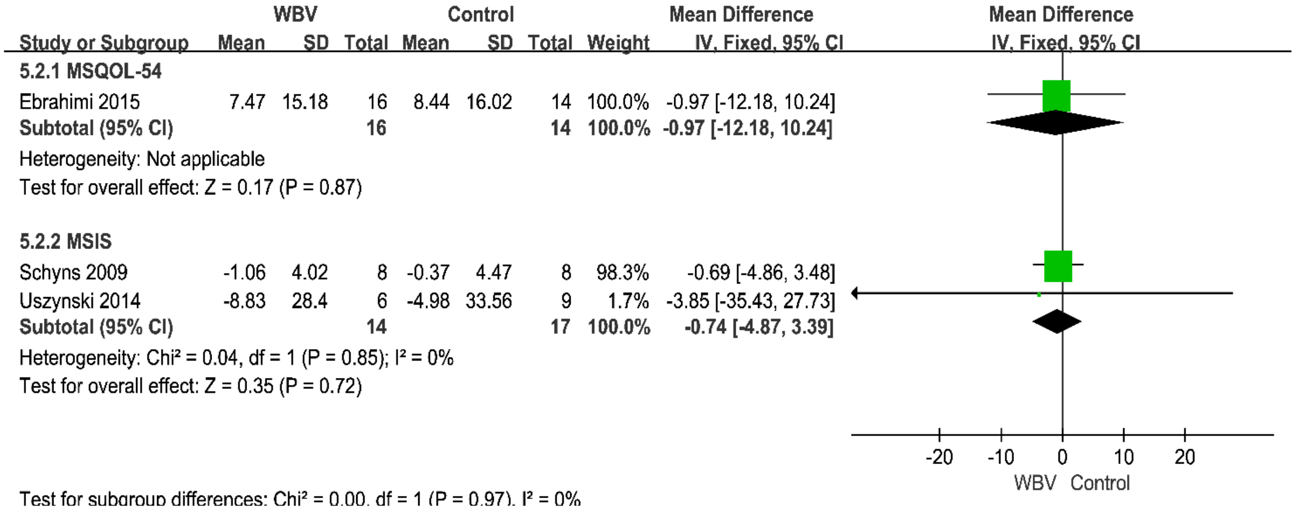

Supplementary Figure 9. The forest plot of health-related quality of life (mentally healthy).

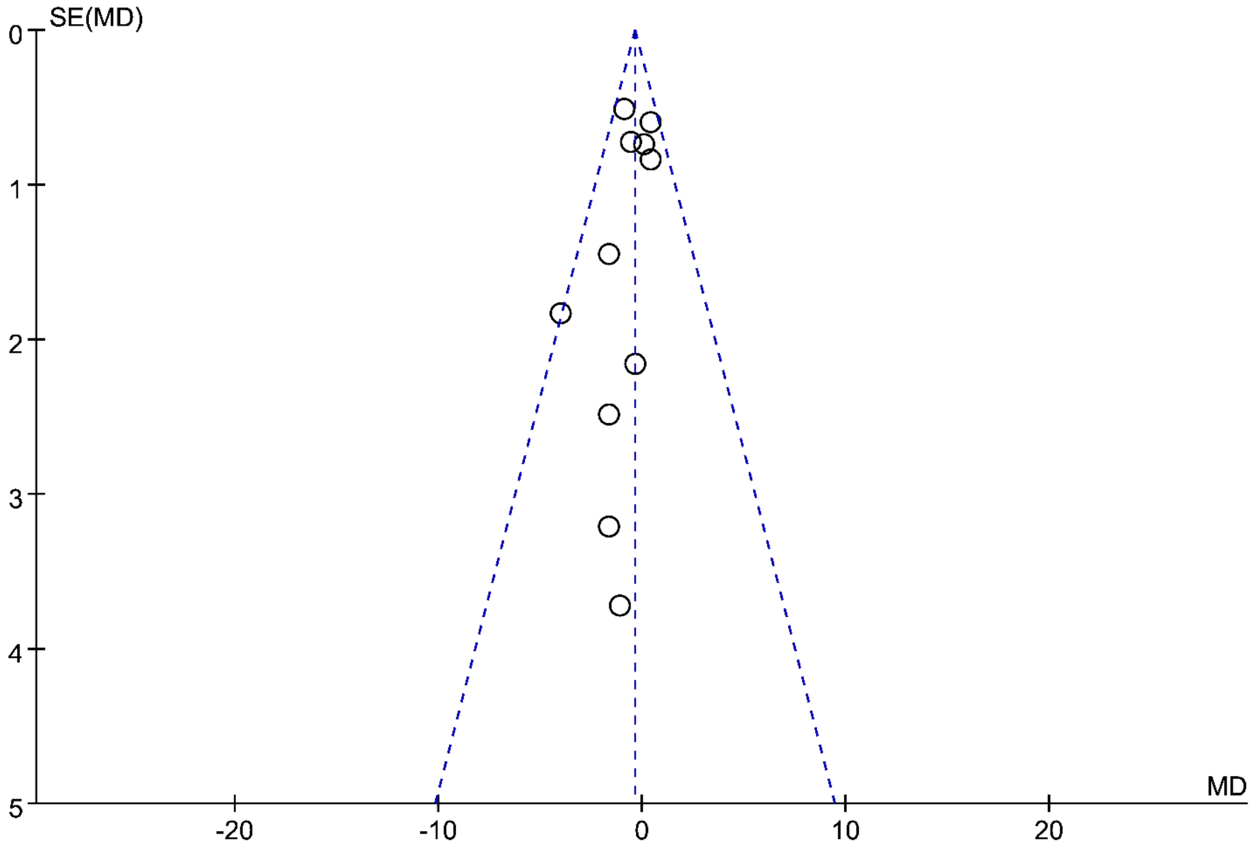

Supplementary Figure 10. The funnel plot of TUG.

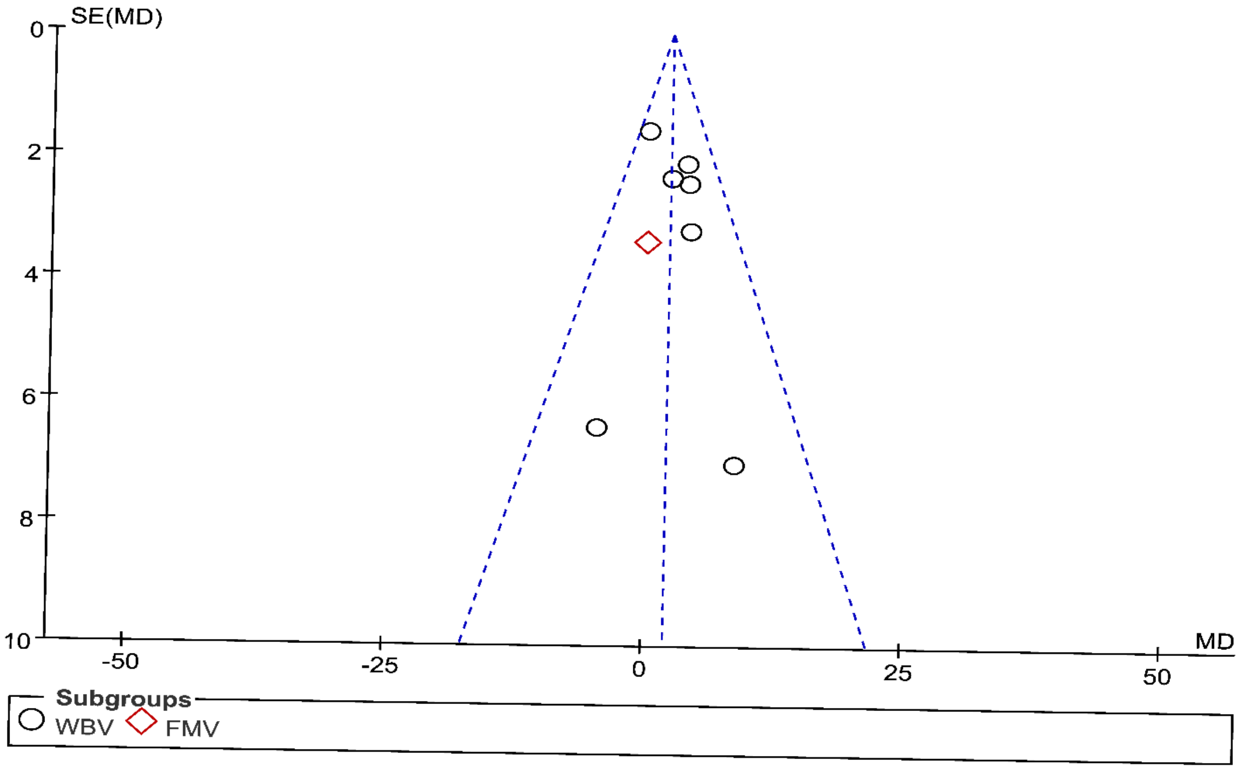

Supplementary Figure 11. The funnel plot of BBS.
