# Supplementary material for: Effects of vibration training on motor and non-motor symptoms for patients with multiple sclerosis: A systematic review and meta-analysis
Source: Front Aging Neurosci. 2022 Aug 5;14:960328. doi: 10.3389/fnagi.2022.960328 (PMC9415382; doi:10.3389/fnagi.2022.960328)
Supplement: Supplementary file 1 [file Data_Sheet_1.ZIP › Supplementary Material/Supplementary_Table.pdf]

Supplementary Material

Supplementary Table 1. Tests for Publication Bias of TUG(Egger's test)

Std_Eff	Coef.	Std. Err.	t	P>|t|	[95% Conf. Interval]	
slope	.4290717	.4273799	1.00	0.342	-.5377288	1.395872
bias	-1.58584	1.083368	-1.46	0.177	-4.036588	.8649083

Supplementary Table 2. Tests for Publication Bias of BBS(Egger's test)

Std_Eff	Coef.	Std. Err.	t	P>|t|	[95% Conf. Interval]	
slope	1.227973	1.039926	1.18	0.282	-1.316634	3.772581
bias	-2.292219	2.607089	-0.88	0.413	-8.671537	4.087099
